# Single-cell landscape of alternative polyadenylation in human lymphoid hematopoiesis

**DOI:** 10.1093/jmcb/mjae027

**Published:** 2024-07-09

**Authors:** Jiaqi Qiang, Shan Yu, Jun Li, Yu Rong, Xiaoshuang Wang, Yong Zhu, Fang Wang

**Affiliations:** State Key Laboratory of Medical Molecular Biology, Department of Biochemistry and Molecular Biology, Institute of Basic Medical Sciences, Chinese Academy of Medical Sciences, School of Basic Medicine, Peking Union Medical College, Beijing 100005, China; The Key Laboratory of RNA and Hematopoietic Regulation, Chinese Academy of Medical Sciences, Beijing 100005, China; Eight-Year Medical Doctor Program, Chinese Academy of Medical Sciences and Peking Union Medical College, Beijing 100730, China; Department of Endocrinology, Key Laboratory of Endocrinology of National Health Commission, Peking Union Medical College Hospital, Chinese Academy of Medical Science and Peking Union Medical College, Beijing 100730, China; State Key Laboratory of Medical Molecular Biology, Department of Biochemistry and Molecular Biology, Institute of Basic Medical Sciences, Chinese Academy of Medical Sciences, School of Basic Medicine, Peking Union Medical College, Beijing 100005, China; The Key Laboratory of RNA and Hematopoietic Regulation, Chinese Academy of Medical Sciences, Beijing 100005, China; Key Laboratory of Digital Technology in Medical Diagnostics of Zhejiang Province, Hangzhou 310030, China; Department of Cardiovascular Medicine, Chongqing Emergency Medical Center, Chongqing University Central Hospital, Chongqing 400014, China; State Key Laboratory of Medical Molecular Biology, Department of Biochemistry and Molecular Biology, Institute of Basic Medical Sciences, Chinese Academy of Medical Sciences, School of Basic Medicine, Peking Union Medical College, Beijing 100005, China; The Key Laboratory of RNA and Hematopoietic Regulation, Chinese Academy of Medical Sciences, Beijing 100005, China; State Key Laboratory of Medical Molecular Biology, Department of Biochemistry and Molecular Biology, Institute of Basic Medical Sciences, Chinese Academy of Medical Sciences, School of Basic Medicine, Peking Union Medical College, Beijing 100005, China; The Key Laboratory of RNA and Hematopoietic Regulation, Chinese Academy of Medical Sciences, Beijing 100005, China; College of Basic Medicine, Chongqing Medical University, Chongqing 400016, China; State Key Laboratory of Medical Molecular Biology, Department of Biochemistry and Molecular Biology, Institute of Basic Medical Sciences, Chinese Academy of Medical Sciences, School of Basic Medicine, Peking Union Medical College, Beijing 100005, China; The Key Laboratory of RNA and Hematopoietic Regulation, Chinese Academy of Medical Sciences, Beijing 100005, China

**Keywords:** alternative polyadenylation, hematopoiesis, lymphoid differentiation, post-transcription, single-cell

## Abstract

Alternative polyadenylation (APA) is an essential post-transcriptional process that produces mature mRNA isoforms by regulating the usage of polyadenylation sites (PASs). APA is involved in lymphocyte activation; however, its role throughout the entire differentiation trajectory remains elusive. Here, we analyzed single-cell 3′-end transcriptome data from healthy subjects to construct a dynamic-APA landscape from hematopoietic stem and progenitor cells (HSPCs) to terminally differentiated lymphocytes. This analysis covered 19973 cells of 12 clusters from five lineages (B cells, CD4^+^ T cells, CD8^+^ T cells, natural killer cells, and plasmacytoid dendritic cells). A total of 2364 genes exhibited differential 3′-untranslated region (3′UTR) PAS usage, and 3021 genes displayed differential intronic cleavage during lymphoid differentiation. We observed a global trend of 3′UTR shortening during lymphoid differentiation. Nevertheless, specific events of both 3′UTR shortening and lengthening were also identified within each cluster. The APA patterns delineated three differentiation stages: HSPCs, precursor cells, and mature cells. Moreover, we demonstrated that the conversion of naïve T cells to memory T cells was accompanied by dynamic APA in transcription factor-encoding genes (*TCF7* and *NFATC2IP*), immune function-related genes (*BCL2, CD5, CD28, GOLT1B*, and *TMEM59*), and protein ubiquitination-related genes (*UBE2G1, YPEL5*, and *SUMO3*). These findings expand our understanding of the underlying molecular mechanisms of APA and facilitate studies on the regulatory role of APA in lymphoid hematopoiesis.

## Introduction

Alternative polyadenylation (APA) is a post-transcriptional regulation for the processing and maturation of mRNA isoforms. In mammalian cells, >70% of genes have multiple polyadenylation sites (PASs). Most PASs are located in 3′-untranslated regions (3′UTRs), generating transcript isoforms that diversify the mRNA stability, localization, and translation rate. Moreover, ∼20% of human genes have intronic PASs, leading to changes in the coding sequence. Recently, advanced technologies, including experimental approaches and computational analyses, have enabled more accurate identification and quantification of PASs (Tian et al., 2007; Derti et al., 2012; Hoque et al., 2013; Singh et al., 2018; Gruber and Zavolan, 2019; Mitschka and Mayr, 2022). Single-cell RNA sequencing (scRNA-seq) based on 3′-tagged data and corresponding APA calculation pipelines has made it possible to portray 3′UTR isoforms at all stages of cell differentiation (Mitschka and Mayr, 2022).

APA is a tissue- and context-specific process (Lianoglou et al., 2013; Mitschka and Mayr, 2022) that has been partially elucidated in the hematopoietic and immune systems. In the early stage of hematopoietic differentiation, 3′UTR shortening is a hallmark of the proliferation of hematopoietic stem cells (HSCs) (Sommerkamp et al., 2020). APA is also a mechanism that is frequently involved in the maturation and activation of both T and B lymphocytes (Sommerkamp et al., 2021). For T cells, global 3′UTR shortening was demonstrated in proliferating T cells (Sandberg et al., 2008; Gruber et al., 2014). For example, due to a switch to a more proximal PAS, the transcription factor NF-ATc is expressed in a shorter form in effector T cells. This shorter form prompts a stronger stimulatory effect on the *IL-2* and *IL-4* promoters, which is necessary for gene induction in effector T cells (Chuvpilo et al., 1999). After T cells are co-stimulated in response to inflammatory reactions or infections, APA is one of the most enriched biological processes (Karginov et al., 2022). For B cells, secretion-coupled APA involving 3′UTR shortening is executed during the differentiation of B cells to plasma cells (Cheng et al., 2020). In addition, the differential usage of intronic PASs in human primary immune cells helps generate truncated proteins during B-cell development (Singh et al., 2018). However, no study has portrayed the dynamic APA that occurs from the top of the hematopoietic hierarchy to terminal-activated lymphocytes.

Here, taking advantage of single-cell transcriptome data for identifying APA events, we aimed to construct a dynamic-APA landscape covering major cell lineages during lymphoid differentiation, including T cells, B cells, natural killer (NK) cells, and plasmacytoid dendritic cells (pDCs). We found that lymphoid differentiation was accompanied by global 3′UTR shortening and enhanced intronic cleavage, with specific APA events occurring within each cluster. The APA patterns helped define differentiation stages and establish temporal sequences. During the peripheral transition from naïve to memory T cells, we observed that PAS switched in functional genes. These findings expand our understanding of the mechanisms involved in active 3′UTR editing and intronic cleavage during lymphoid differentiation and have important physiological implications in the context of lymphoid hematopoiesis.

## Results

### Identification of credible PAS during lymphoid differentiation

**Figure 1:**
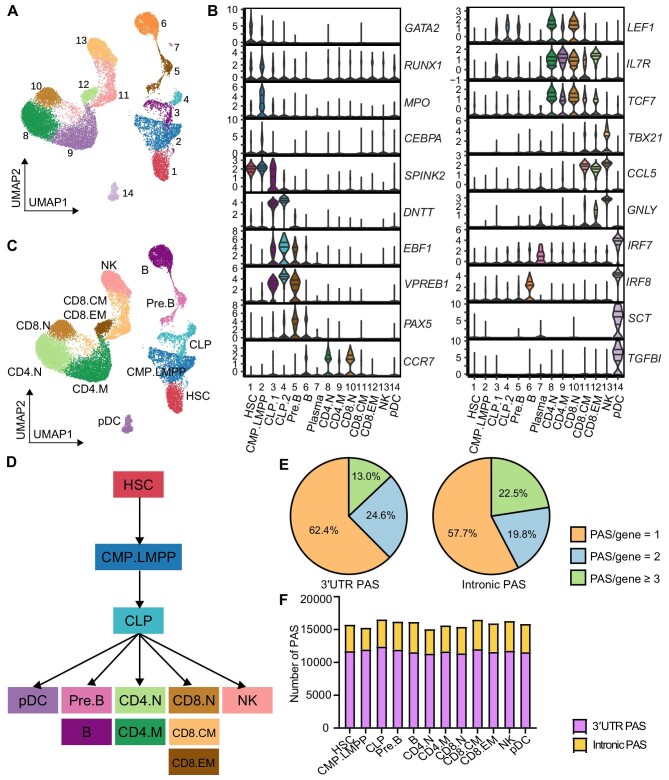
Single-cell clustering and PAS identification during lymphoid differentiation. (**A**) Reclustering-generated 14 cell populations during lymphoid differentiation, visualized on a UMAP plot. (**B**) Violin plots showing the expression levels of HSPC and lymphoid cell marker genes in each population. (**C**) The final single-cell clustering used in the subsequent analysis. (**D**) Schematic illustration of the lymphoid differentiation trajectory. (**E**) Pie charts of genes with single and multiple PASs during lymphoid differentiation. PAS/gene refers to the number of 3′UTR or intronic PASs per gene. (**F**) The number of 3′UTR and intronic PASs across all lymphoid cell populations.

To assemble a comprehensive atlas of hematopoietic stages and lineages, we analyzed a set of 3′-tagged scRNA-seq data from bone marrow and peripheral blood mononuclear cells from healthy donors (Granja et al., 2019). Through reclustering, we identified 14 distinct clusters (Figure 1A). These clusters were identified using a set of marker genes that were manually curated (Figure 1B). We combined the two subclusters of common lymphoid progenitors (CLPs) into one cluster and removed the plasma cell cluster with <100 cells. As a result, we focused our analysis on 12 clusters comprising a total of 19973 cells (Figure 1C) and delineated the differentiation path of these clusters into five lymphoid lineages (B cells, CD4^+^ T cells, CD8^+^ T cells, NK cells, and pDCs) based on the literature (Figure 1D; Murphy and Weaver, 2016; Buenrostro et al., 2018; Amon et al., 2019).

To identify and quantify PAS usage at the single-cell level, we used the 3′-read-enrichment-based scAPA pipeline (Shulman and Elkon, 2019). A total of 15827 3′UTR PASs and 4328 intronic PASs located in 10176 protein-coding genes were enriched during lymphoid differentiation. We then investigated how the identified PASs were distributed across genes and cell clusters. The proportions of genes with multiple PASs in the 3′UTR (37.6%) and introns (42.3%) were similar (Figure 1E). The number of identified 3′UTR PAS was ∼2–3 times that of intronic PAS, and each cell cluster had a comparable proportion of 3′UTR and intronic PAS (Figure 1F). For both 3′UTR PAS and intronic PAS, the number and proportion of PAS per gene were roughly equivalent across all cell clusters, with the exception that the absolute number of intronic PAS in common myeloid progenitor/lymphoid-primed multipotent progenitor (CMP.LMPP) cells was relatively smaller than those of other cell clusters (Supplementary Figure S1A). These results suggest that the proportion of genes undergoing APA is probably steady during lymphoid differentiation.

Quality control was performed to confirm the reliability of the identified PASs. We compared the enriched 3′UTR PASs with known sites in the PolyA_DB database (version 3.2; Wang et al., 2018) and found that 74.15% of them were located within 100 nucleotides (nt) of known exonic PASs (Supplementary Figure S1B). The 3′-end of up to 82.3% of the 3′UTR PASs was enriched near the canonical poly(A) signals AAUAAA and AUUAAA (Supplementary Figure S1C and D; Tian and Graber, 2012; Gruber and Zavolan, 2019; Shulman and Elkon, 2019). These findings indicate that the identified 3′UTR PAS is accurate and credible during lymphoid differentiation.

In the intronic PAS, the motifs AAAAAAAA and AAAAAUAA were found to be more enriched compared to the canonical signals. The canonical signals were located ∼100 nt downstream of the identified PAS, as shown in Supplementary Figure S1C and D. However, it is important to note that these peaks may potentially be unreliable due to the presence of internal priming artifacts, as they were in close proximity to A-rich regions. Therefore, we focused mainly on APA in the 3′UTR in subsequent analyses and experiments to avoid confusion or misleading results.

### Global 3′UTR shortening during lymphoid differentiation

**Figure 2:**
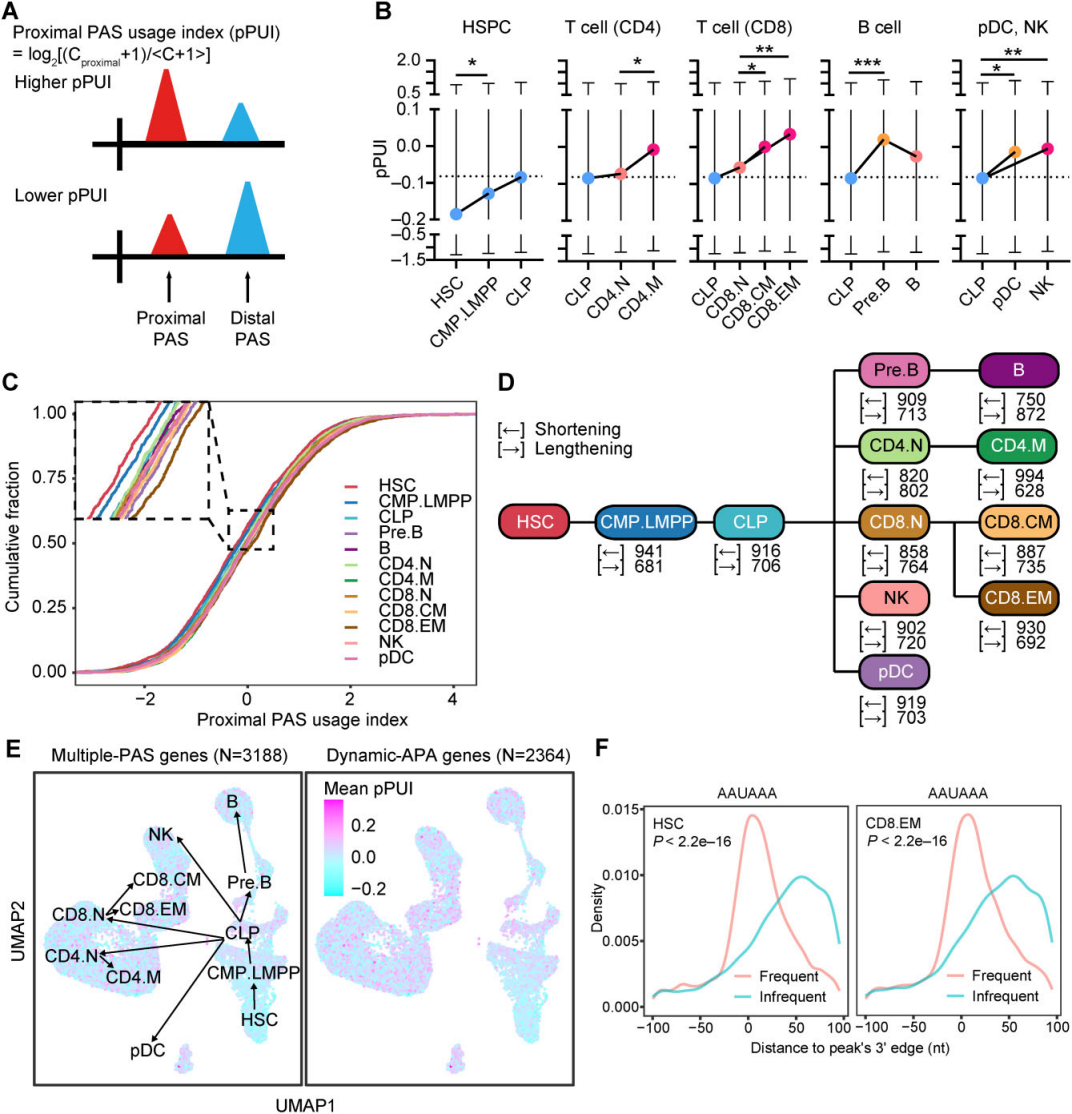
Global APA analysis reveals a trend toward 3′UTR shortening during lymphoid differentiation. (**A**) Schematic illustration of the pPUI calculation. C_proximal_ is the read count of the proximal peak, and 〈C + 1〉 is the geometric mean of the counts of all the peaks associated with the 3′UTR. (**B**) Average pPUI of the 3188 multiple-PAS genes in each lymphoid cell population. The *t*-test was performed to determine the difference in pPUI between adjacent populations in the differentiation trajectory. *P < 0.05; **P < 0.01; ***P < 0.001. (**C**) Cumulative distribution curve of the pPUI of 2364 dynamic-APA genes in 12 lymphoid cell populations. The square on the upper left shows partial magnification. The statistical significance between adjacent clusters during lymphoid differentiation was as follows: HSC vs. CMP.LMPP, *P* = 0.065; CMP.LMPP vs. CLP, *P* = 0.098; CLP vs. Pre.B, *P* = 0.0008; CLP vs. CD4.N, *P* = 0.591; CLP vs. CD8.N, *P* = 0.207; CLP vs. NK, *P* = 0.012; CLP vs. pDC, *P* = 0.022; Pre.B vs. B, *P* = 0.128; CD4.N vs. CD4.M, *P* = 0.027; CD8.N vs. CD8.CM, *P* = 0.091; CD8.N vs. CD8.EM, *P* = 0.003. (**D**) A schematic diagram illustrating the number of 3′UTRs containing two PASs that undergo shortening or lengthening between adjacent populations during the process of lymphoid differentiation. (**E**) The average pPUI of multiple-PAS genes (left) or dynamic-APA genes (right) was calculated at the single-cell level and projected onto the transcriptome clustering map. (**F**) The density distribution of the poly(A) signal AAUAAA in proximity to frequent (with the highest expression) and infrequent (with the lowest expression) PASs of 3′UTR exhibiting dynamic-APA events in HSCs and CD8.EM cells, respectively. The Kolmogorov–Smirnov test was used to demonstrate the distribution difference between frequent PASs and infrequent PASs in the region located 100 nt upstream and 100 nt downstream from the 3′ edge of the identified PAS peak.

Having identified credible 3′UTR PASs, we next aimed to portray the global dynamic APA during lymphoid differentiation. The proximal PAS usage index (pPUI) was calculated to quantify differential 3′UTR PAS usage during cell differentiation. A higher pPUI value indicated a preference for the proximal PAS, whereas a lower value indicated a preference for the distal PAS (Figure 2A). Among the 3188 genes with >1 PAS (termed ‘multiple-PAS genes’), we identified 2364 genes showing differential 3′UTR PAS usage among the lymphoid cell clusters (termed ‘dynamic-APA genes’).

During lymphoid differentiation, the 3188 multiple-PAS genes exhibited a gradual increase in pPUI. However, an exception was observed in the B cell lineage (Figure 2B). The hematopoietic stem and progenitor cell (HSPC) populations had more dynamic-APA genes with a preference for distal PASs, whereas the terminal populations exhibited a preference for proximal PASs (Figure 2C). Moreover, by comparing adjacent clusters along the lymphoid differentiation path, we observed a greater extent of 3′UTR shortening in mature cell lineages, except for the Pre.B to B cell transition (Figure 2D). These findings suggest that a greater proportion of genes experience 3′UTR shortening during lymphoid differentiation.

We then examined PAS usage at the single-cell level. Within each cluster, cells showed consistent PAS preference for both multiple-PAS genes and dynamic-APA genes. Along the lymphoid differentiation path, an increasing number of cells opted for the proximal 3′UTR PAS, leading to the production of transcripts with shortened 3′UTRs (Figure 2E). This phenomenon suggests that 3′UTR shortening is closely related to the differentiation and maturation of most lymphoid cells.

To explore how cells determine, which 3′UTR PAS to choose, we selected HSCs and CD8^+^ effector memory T (CD8.EM) cells as representative clusters for analysis. As illustrated in Figure 2B and C, HSCs exhibited a preference for distal PASs, whereas CD8.EM cells exhibited a preference for proximal PASs. Motif enrichment analysis showed that, in both HSCs and CD8.EM cells, the poly(A) signal AAUAAA and the PAS enhancer UGUA were significantly enriched around the frequent PAS (defined as the most highly expressed PAS in each 3′UTR) (Figure 2F; Supplementary Figure S2). This suggests that regulatory sequences surrounding the PAS probably influence PAS selection, irrespective of its proximity.

### Lymphoid cell-specific APA events in the 3′UTR

**Figure 3:**
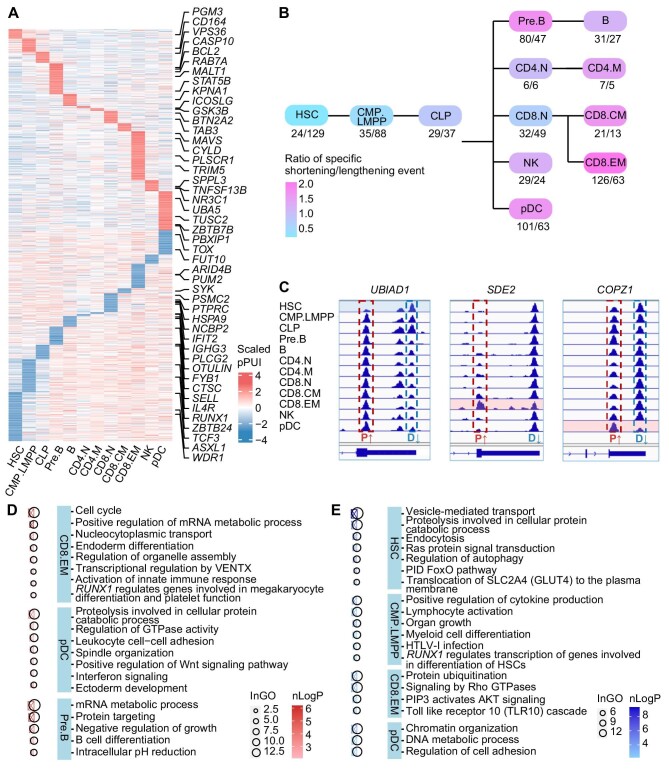
Specific 3′UTR shortening/lengthening in each cell population during lymphoid differentiation. (**A**) pPUI heatmap of genes with specific 3′UTR shortening/lengthening in the 12 lymphoid cell populations. Higher pPUI indicates specifically shortened 3′UTR, while lower pPUI indicates specifically lengthened 3′UTR. For populations in which the number of cell type-specific APA events was insufficient for GO enrichment, genes associated with lymphoid hematopoiesis and immune function in health and disease are labeled on the right. (**B**) Schematic illustration of the ratio of specific 3′UTR shortening/lengthening in each population. (**C**) Case visualization of cell type-specific APA events. Left, *UBIAD1* exhibits 3′UTR lengthening in HSCs and shortening in terminally differentiated cells; middle, *SDE2* exhibits 3′UTR shortening in CD8.EM cells; right, *COPZ1* exhibits 3′UTR shortening in pDCs. P, proximal end of the 3′UTR; D, distal end of the 3′UTR. (**D**) GO enrichment analysis of cell type-specific 3′UTR-shortening APA events in CD8.EM cells, pDCs, and Pre.B cells. (**E**) GO enrichment analysis of cell type-specific 3′UTR-lengthening APA events in HSCs, CMP.LMPP cells, CD8.EM cells, and pDCs.

After characterizing the global trend of APA during lymphoid differentiation, we were curious about how APA events are expressed and function within each specific lineage. We first identified cell type-specific shortening and lengthening of the 3′UTR in each lymphoid cluster (Figure 3A). As shown in the trajectory map, the ‘ratio of specific shortening/lengthening events’, which compares the number of APA events that undergo 3′UTR shortening to the number of those undergo 3′UTR lengthening, increased as the lineages progressed toward terminal differentiation, with the exception of B cells (Figure 3B). These findings indicate that in the stem/progenitor stage, the longer form of 3′UTR is probably more important, while in the terminal stage, the shorter form of 3′UTR becomes more important.

Three specific APA events, characterized by the utilization of the most specific 3′UTR PAS, were visually represented (Figure 3C). *UBIAD1* preferred the distal 3′UTR in HSCs and the proximal 3′UTR in terminally differentiated cells [B cells, CD4^+^ memory T (CD4.M) cells, CD8^+^ central memory T (CD8.CM) cells, CD8.EM cells, NK cells, and pDCs]. This gene has a *Drosophila* ortholog that is pivotal for both hemocyte proliferation and differentiation (Xia et al., 2015). In turn, *SDE2* exhibited a preference for the proximal PAS in CD8.EM cells. This gene has been reported as an alternative splicing regulator (Floro et al., 2021) that is overexpressed in peripheral blood mononuclear cells and lymph nodes (https://www.genecards.org/cgi-bin/carddisp.pl?gene=SDE2#expression). *COPZ1* has a specifically shortened 3′UTR in pDCs and is associated with immune cell infiltration and pro-inflammatory cytokines in tumor cells (Hong et al., 2023). Correlation analyses revealed that the expression level of *UBIAD1* increased with the preference for the distal PAS in HSCs (*P*  < 0.05). For *SDE2* and *COPZ1*, their expression levels were correspondingly elevated in the clusters in which they had a specific preference for the proximal PAS (*P*  < 0.05). Although there is no evidence directly showing the cell type-specific functions of these genes, our findings suggest that they may play potentially significant roles in specific lymphoid clusters.

To further elucidate the functions of cell type-specific APA, we performed Gene Ontology (GO) enrichment analysis. Among the clusters exhibiting significant APA events, CD8.EM cells demonstrated the most pronounced shortening of 3′UTRs, which exhibited significant associations with cellular processes such as the cell cycle, mRNA metabolism, activation of the innate immune response, etc. pDCs had the second largest number of 3′UTR-shortening genes that were involved in proteolysis, GTPase activity, leukocyte cell–cell adhesion, interferon signaling, etc. In Pre.B cells, 3′UTR shortening was associated with mRNA metabolism, B cell differentiation, intracellular pH reduction, etc. (Figure 3D). HSCs had the largest number of genes with specifically lengthened 3′UTRs, which were involved in vesicle-mediated transport, proteolysis, Ras protein signal transduction, etc. Interestingly, in the CMP.LMPP progenitor cluster, we observed several 3′UTR lengthening events related to lymphoid function, including positive regulation of cytokine production, lymphocyte activation, human T-lymphotropic virus type 1 infection, and HSC differentiation regulated by *RUNX1* (Figure 3E). The number of cell type-specific APA events in the remaining populations was insufficient for the performance of the GO enrichment analysis. Genes associated with lymphoid hematopoiesis and immune function in health and disease were labeled (Figure 3A), and the detailed functional annotations are presented in Supplementary Table S3.

### 3′UTR APA patterns are associated with lymphoid differentiation stages

**Figure 4:**
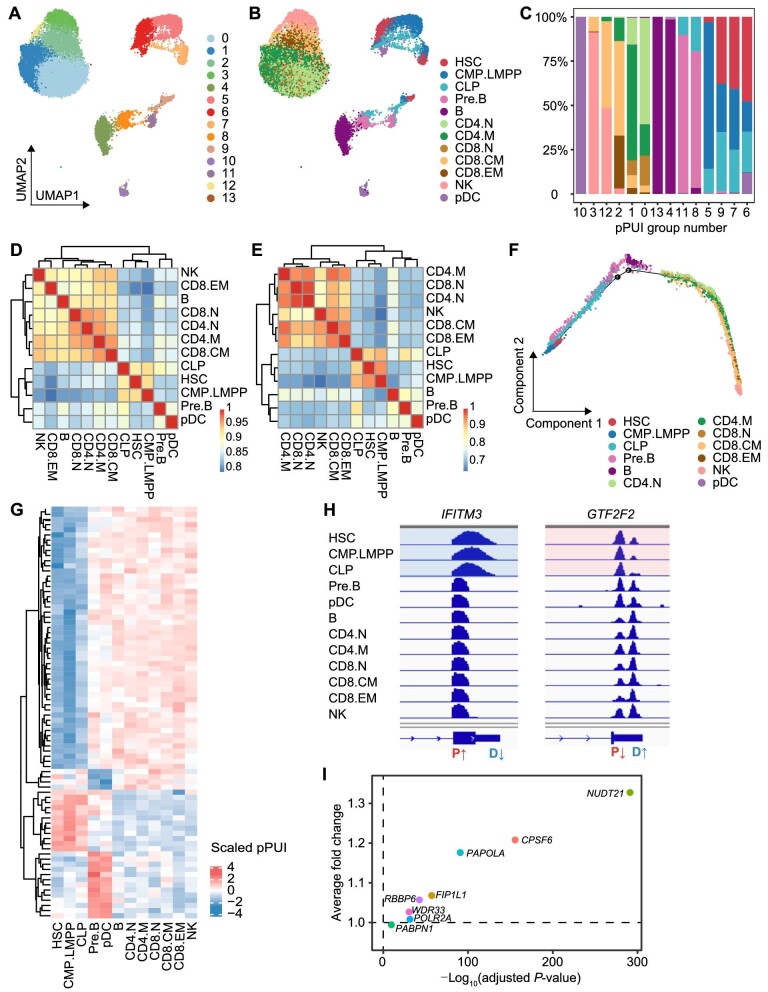
3′UTR APA patterns reflect lymphoid differentiation stages. (**A**) Single-cell clustering using the pPUI expression matrix-generated 14 populations. (**B**) pPUI single-cell clustering labeled with cell clusters based on RNA expression profiles. (**C**) The composition of RNA-based cell clusters within each pPUI-based cluster. (**D**) Pearson's correlation and clustering analyses of the pPUI expression matrix of the 12 populations. (**E**) Pearson's correlation and clustering analyses of RNA expression profiles of the 12 populations. (**F**) Pseudotime analysis showing the differentiation trajectory based on RNA expression profiles. (**G**) Heatmap displaying stage-specific APA events based on pPUI values. Higher pPUI indicates specifically shortened 3′UTR, while lower pPUI indicates specifically lengthened 3′UTR. (**H**) Visual representation of stage-specific APA genes. Left: *IFITM3*, a gene with a lengthened 3′UTR in the HSPC stage; right: *GTF2F2*, a gene with a shortened 3′UTR in the HSPC stage. (**I**) Differential expression analysis of eight APA core regulators between HSPCs (HSCs, CMP.LMPP cells, and CLP cells) and mature lymphocytes (NK, CD8.EM, B, CD8.N, CD4.N, CD4.M, and CD8.CM cells). Vertical dashed line, *P* = 0.05; horizontal dashed line, average fold change = 1.

Because APA exhibited a unique pattern in each lymphoid cluster, we examined whether APA patterns were associated with differentiation stages. We first reclustered the single cells using a pPUI expression matrix and generated 14 groups (Figure 4A), which resembled the distribution of the original clusters based on RNA expression profiles (Figure 4B). In the clustering map based on the pPUI expression matrix, closely located groups had similar compositions of two or three original clusters. Notably, clusters belonging to the same pPUI group tended to be contiguous in the differentiation trajectory (Figure 4C). These findings suggest that APA patterns could potentially serve as indicators of the stage of lymphoid differentiation.

Consequently, we wondered whether APA patterns could help define different differentiation stages. According to the pPUI expression matrix, the lymphoid clusters could be aggregated into three stages: HSPC (HSC, CMP.LMPP cells, and CLP cells), precursor cells (Pre.B cells and pDCs), and mature cells [NK cells, CD8.EM cells, B cells, CD8^+^ naïve T (CD8.N) cells, CD4^+^ naïve T (CD4.N) cells, CD4.M cells, and CD8.CM cells] (Figure 4D). This classification was similar to the cluster aggregation based on RNA expression profiles. The only difference was that B cells were classified as mature cells at the pPUI level, whereas they were classified as precursor cells at the RNA level (Figure 4E). This APA-based stage classification was consistent with the pseudo-chronological order. Moreover, it is worth mentioning that the pDC cluster exhibited relatively advanced status in the trajectory, similar to the status of the Pre.B cluster (Figure 4F; Supplementary Figure S3A). These findings confirm that 3′UTR APA patterns reflect the lymphoid status in the trajectory to a certain extent.

Because APA patterns showed similarities within each differentiation stage, we next investigated stage-specific 3′UTR APA events (Figure 4G). The majority of genes in the HSPC cluster had longer forms of 3′UTRs, which were associated with functions such as RNA metabolism, negative regulation of catalytic activity, immune system development, etc. (Supplementary Figure S3B). Among these genes, *IFITM3* encodes an interferon-induced antiviral protein (Lee et al., 2020), showing typical APA changes (Figure 4H). Other genes related to the immune system included *RHOH, ZFP36L2, GPS2, TNFAIP3*, etc. Interestingly, the transcription factor-encoding gene *GTF2F2* exhibited synchronous APA changes with the differentiation stage. It predominantly used a proximal PAS in HSPCs, approximately equal proximal and distal PAS in precursor cells, and shifted to a distal PAS in mature lymphocytes (Figure 4H). In addition to its role in forming a pre-transcriptional initiation complex (Zhang et al., 2022), the involvement of *GTF2F2* in lymphoid differentiation deserves further exploration.

To elucidate the underlying mechanism, we then investigated the RNA expression levels of core APA regulators from major functional clusters (Tian and Manley, 2017; Mitschka and Mayr, 2022) in the lymphoid populations. Intriguingly, the expression patterns of the eight most highly expressed APA regulators were similar, with relatively low expression levels in HSPCs and high expression levels in mature cells (Figure 4I; Supplementary Figure S3C). This is consistent with previous reports stating that during differentiation, the expression levels of APA regulators are elevated in association with a preference for the proximal PAS (Ji et al., 2009; Ji and Tian, 2009).

### Dynamic 3′UTR APA events in the peripheral differentiation of T cells

**Figure 5:**
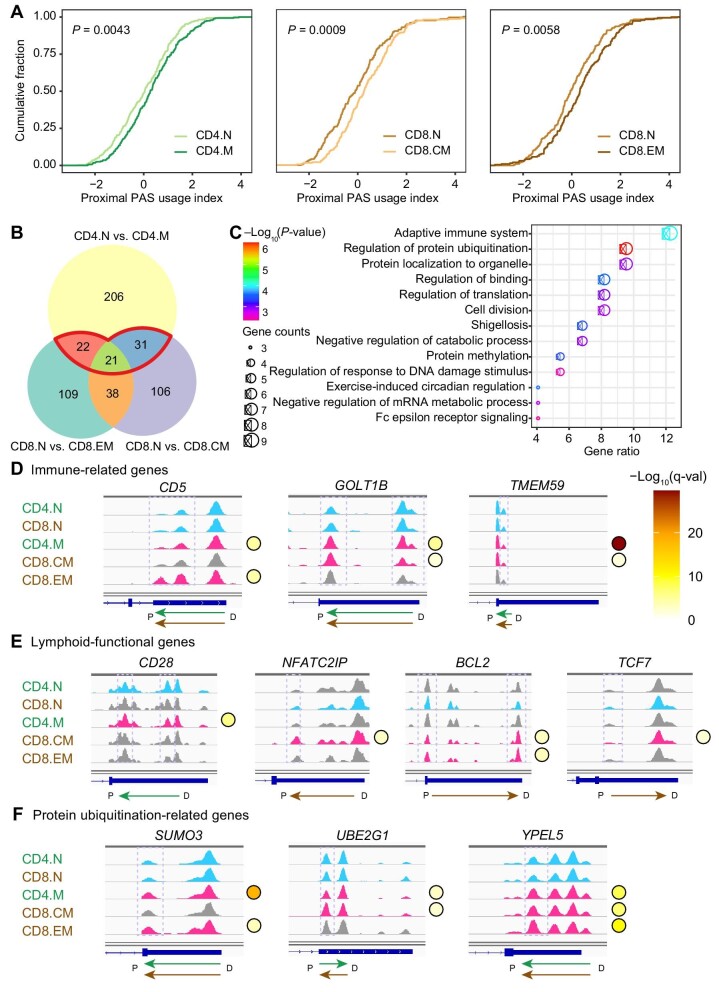
Differential 3′UTR APA analysis during the peripheral differentiation of T cells. (**A**) Cumulative distribution curve of the pPUI values of dynamic-APA genes during peripheral T cell differentiation. The Kruskal–Wallis test was used to determine the statistical significance of differences. Left, CD4.N vs. CD4.M; middle, CD8.N vs. CD8.CM; right, CD8.N vs. CD8.EM. (**B**) Venn plot showing the differential APA events between naïve and memory T cells. The gene set of interest is outlined in red. (**C**) GO enrichment analysis of the gene set of interest. (**D**–**F**) Case visualization of differential APA events related to immunological function (**D**), lymphocyte function (**E**), and protein ubiquitination (**F**) during peripheral differentiation. q-val, adjusted *P*-value. Purple dashed box, PAS with significant change; blue coloring, clusters with significant PAS usage in the naïve T cell stage; pink coloring, clusters with significant PAS usage in the memory T cell stage; green arrow indicates the trend of APA change in the CD4^+^ T cell lineage; brown arrow indicates the trend of APA change in the CD8^+^ T cell lineage.

In addition to central differentiation, peripheral activation is also an essential process for the immune function of lymphocytes. When stimulated by antigens or other environmental factors, a proportion of naïve T cells switch to memory T cells as part of an immune response (Sprent and Surh, 2011). We were curious about the role of dynamic 3′UTR APA in this process. During the conversion of CD4.N cells to CD4.M cells, CD8.N cells to CD8.CM cells, and CD8.N cells to CD8.EM cells, we observed 3′UTR shortening (Figure 5A). We then identified differential APA events between naïve and memory T cells. By intersecting the CD4.N versus CD4.M comparison with the union of CD8.N versus CD8.CM and CD8.N versus CD8.EM, a total of 74 genes were identified as exhibiting mutual dynamic-APA events in both CD4^+^ T cells and CD8^+^ T cells (Figure 5B). These genes were functionally enriched in acquired immunity, protein ubiquitination, cell division, the Fc epsilon receptor signaling pathway, etc. (Figure 5C).

Several immune-related genes were found during the peripheral differentiation of both CD4^+^ and CD8^+^ T cells (Figure 5D). As naïve cells differentiated to memory cells, the 3′UTR of *CD5, GOLT1B*, and *TMEM59* was shortened. *CD5* encodes a cysteine-rich domain scavenger receptor that is present on the surface of T cells and B cells and acts to regulate T cell proliferation. *GOLT1B* encodes the Golgi transport 1B protein that is involved in the positive regulation of IκB kinase/NF-κB signaling (https://www.ncbi.nlm.nih.gov/gene/51026#summary). *GOLT1B* upregulates PD-L2 and promotes T lymphocyte apoptosis in the tumor microenvironment (Liu et al., 2021). *TMEM59* encodes transmembrane protein 59 that regulates autophagy in response to bacterial infection (https://www.ncbi.nlm.nih.gov/gene/9528).

Several critical lymphoid functional genes exhibited significant 3′UTR changes in only one lineage of CD4^+^ or CD8^+^ T cells (Figure 5E). During the conversion of CD4.N cells to CD4.M cells, the usage of the *CD28* proximal PAS was increased. *CD28* is critical for T cell proliferation and survival, cytokine production, and CD4^+^ T cell activation, and its persistent expression is necessary for CD4^+^ T cell polarization in response to infection (Linterman et al., 2014). During the conversion of CD8.N cells to CD8.CM cells, the 3′UTR of *NFATC2IP* was shortened, consistent with previous reports. The protein encoded by *NFATC2IP* can promote Pol II transcription and regulate the transcription of specific cytokines in T cells. The two longer transcripts of *NFATC2IP* expressed in naïve T cells switch to a shorter one in effector T cells to ensure rapid accumulation of the protein product (Chuvpilo et al., 1999). For genes with 3′UTR lengthening, *BCL2* used the distal PAS in both CD8.CM and CD8.EM cells. The protein encoded by *BCL2* is an apoptosis regulator. The relatively high expression of *BCL2* leads to the memory function of CD8^+^ T cell lineage (Dunkle et al., 2013). *TCF7* used the distal PAS in CD8.CM cells to encode a transcription factor required for the production and maintenance of CD8.CM cells (Zhang et al., 2021).

Notably, we detected protein ubiquitination-related genes that also underwent significant 3′UTR shortening during the peripheral differentiation of both CD4^+^ and CD8^+^ T cells, including *SUMO3, UBE2G1*, and *YPEL5*, with the exception of *UBE2G1* in CD4^+^ T cells (Figure 5F). Protein ubiquitination is a reversible covalent modification process that plays an important role in various aspects of the immune system, including the regulation of T cell differentiation and activation, thereby maintaining efficient adaptive immune responses against pathogens and self-organization immune tolerance (Hu and Sun, 2016). The dynamic-APA events of these genes may play a regulatory role in the process of lymphocytic protein ubiquitination, and the underlying functional mechanism is worthy of further exploration.

Given the subtle changes observed in some cases, we further explored the differences in pPUI at the single-cell level. Consistent with the differential analysis of peak counts at the population level, during the transition from CD4.N to CD4.M cells and CD8.N to CD8.CM cells, the pPUI of the *TMEM59* mRNA was significantly increased (Supplementary Figure S4A), indicating the shortening of the *TMEM59* 3′UTR, whereas the pPUI of the *TCF7* mRNA was decreased (Supplementary Figure S4B), suggesting the lengthening of its 3′UTR. In addition to these lymphoid-associated genes, we also identified several cases with significant peak changes (Supplementary Figure S4C–E). Nevertheless, these cases were not included in the subsequent validation study, as they were less directly pertinent to peripheral T cell function.

### Experimental validation of dynamic 3′UTR APA events in CD8^+^ T cells

**Figure 6:**
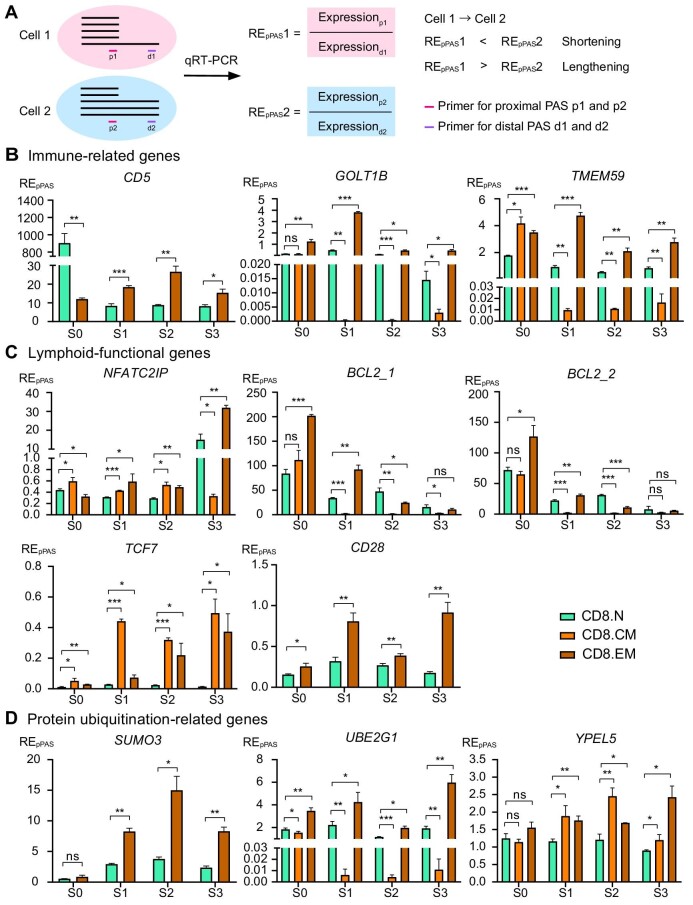
*In vitro* validation of the functional 3′UTR PAS switch in peripheral CD8^+^ lymphocytes. (**A**) Schematic illustration of the comparison of RE_pPAS_ between different clusters. p1, proximal PAS in cell 1; p2, proximal PAS in cell 2; d1, distal PAS in cell 1; d2, distal PAS in cell 2. (**B**) RE_pPAS_ of the immune-related genes *CD5, GOLT1B*, and *TMEM59*. (**C**) RE_pPAS_ of the lymphoid functional genes *NFATC2IP, BCL2, TCF7*, and *CD28*. (**D**) RE_pPAS_ of the protein ubiquitination-related genes *SUMO3, UBE2G1*, and *YPEL5*. *P < 0.05, **P < 0.01, ***P < 0.001.

We then validated the dynamic 3′UTR APA events in peripheral CD8^+^ T cell differentiation in healthy human donors by isolating the CD8.N, CD8.CM, and CD8.EM cells from peripheral blood. The comparison of the relative expression of the proximal PAS (RE_pPAS_) was used to quantify the dynamic 3′UTR changes between different cell clusters (Figure 6A). Among the genes associated with immunological functions, we observed 3′UTR shortening in *CD5, GOLT1B*, and *TMEM59* during the conversion of CD8.N to CD8.EM cells, which was consistent with the sequencing results. In contrast to the sequencing results, during the differentiation from CD8.N to CD8.CM cells, the 3′UTRs of *GOLT1B* and *TMEM59* were lengthened rather than shortened (Figure 6B). Regarding the CD8^+^-specific dynamic-APA events of lymphoid functional genes, as expected, the 3′UTR of *NFATC2IP* was shortened and that of *BCL2* was lengthened, which validated their significant 3′UTR PAS changes during CD8^+^ T cell differentiation. In contrast to the sequencing results, the 3′UTR of *TCF7* was shortened from CD8.N to both CD8.CM and CD8.EM cells, rather than lengthened in CD8.CM cells, suggesting that the trend of APA might need further exploration. Interestingly, we also found that the 3′UTR of *CD28* was shortened during the differentiation from CD8.N to CD8.EM cells, indicating that this gene is probably functional in both CD8^+^ and CD4^+^ T cell differentiation. The analysis of the sequencing data also revealed that the expression of *CD28* increased along the peripheral differentiation in both CD8^+^ and CD4^+^ T cell lineages. This result suggests that this gene encodes protein that is essential for T cell proliferation and survival (Figure 6C). Among the protein ubiquitination-related genes, *SUMO3, UBE2G1*, and *YPEL5* exhibited 3′UTR shortening during the differentiation from CD8.N to CD8.EM cells. 3′UTR shortening of *YPEL5* was also discovered in CD8.CM cells as expected, whereas 3′UTR lengthening instead of shortening was found in *UBE2G1* (Figure 6D).

### Changes in intronic APA during lymphoid differentiation

In addition to investigating dynamic APA in the 3′UTR, we explored the trend of dynamic APA in intronic regions during lymphoid differentiation. The comparison of the intronic PAS usage index (iPUI) between two cell clusters revealed that a higher iPUI value indicated a preference for intronic PAS, whereas a lower iPUI value indicated a preference for 3′UTR PAS. In fact, 3021 genes had significantly altered iPUI values among these lymphoid clusters. Compared with HSCs, the CMP.LMPP cell cluster had a higher number of intronic cleaved genes with attenuated expression, whereas the CLP cluster had a higher number of intronic cleaved genes with enhanced expression. The 3021 genes that exhibited significant intronic APA showed a gradual increase in iPUI as HSPCs differentiated into mature blood cells with the exception of the CD8^+^ T cell lineage (Supplementary Figure S5A). HSPC populations had more dynamic-APA genes with a preference for 3′UTR PASs; in turn, the terminal populations preferred intronic PASs (Supplementary Figure S5B). Next, we identified 1399 cell type-specific intronic cleavage events for each lymphoid cluster. In general, there were more attenuated events than enhanced events (Supplementary Figure S5C and D). We also detected 95 specific intronic APA events in the HSPC stage (Supplementary Figure S5E). These findings indicate that as stem/progenitor cells transition to terminally differentiated cells, transcripts in the intronic form may also play an increasingly important role.

## Discussion

In this research, we demonstrated the APA landscape during human lymphoid differentiation at the single-cell level. APA, a key post-transcriptional mechanism, generates distinct transcripts that play different roles in human health and disease (Gruber and Zavolan, 2019). While previous studies have elucidated tissue-specific PASs in the blood (Singh et al., 2018; Yang et al., 2022), this study represents the first demonstration of PASs across populations encompassing all lymphoid lineages. Because there are currently few PAS annotations for blood and immune cells in publicly available databases, our study provides a resource for future APA studies in both healthy individuals and patients with lymphatic system disorders.

3′UTR shortening has been observed in the terminal differentiation stage of lymphocytes, including proliferating T cells (Shulman and Elkon, 2019) and secretory B cells (Cheng et al., 2020); however, studies focusing on the 3′UTR changes at the HSPC stage are still lacking. This study filled this gap by revealing the picture of the global 3′UTR shortening trend along the lymphoid differentiation, from the most primitive stage to the end of this process. It is worth mentioning that, among the five lymphoid lineages, only the differentiation from the Pre.B to the B cell cluster exhibited 3′UTR lengthening instead of shortening. Lengthened 3′UTRs were previously observed in neuronal differentiation and cancer cells enriched for certain functional groups (Tian and Manley, 2017). The lengthening of 3′UTRs in B cells may ensure the expression of genes essential for survival. Interestingly, secretion-coupled 3′UTR shortening was reported in the transformation of B cells into plasma cells (Cheng et al., 2020). However, due to the limited number of cells in the plasma cell cluster in our study, this observation cannot be reliably verified.

Enabled by more efficient experimental and computational methods of capturing PAS in diverse single-cell types, mounting evidence shows that APA is involved in cellular regulation and function (Ye et al., 2020). Using transcriptomic data at single-cell resolution, we discovered specific dynamic-APA events for each cell cluster. Some of these APA events were associated with transcription and RNA metabolism, whereas others were associated with the biological function of lymphocytes. Nevertheless, the functional role of drastically switched PAS in lymphoid differentiation warrants further exploration. Another novel finding was that APA patterns may contribute to a more precise characterization of the differentiation stage. The APA patterns were similar in cell clusters that were timely adjacent in the differentiation trajectory. Based on the analysis reported in this study, a possible underlying mechanism was that the preference for proximal or distal PAS was determined by PAS signals and APA regulators. However, it remains to be discovered whether APA is regulated to mediate cell-state changes or APA patterns are the consequences of cell-state changes.

In the peripheral blood, naïve T cells differentiate into effector T cells after stimulation. Instead of becoming involved in lymphocyte recycling, effector T cells generally migrate to peripheral inflammation sites or lymphoid tissues, to exert their effects, and have a short survival period. Some of them differentiate into memory T cells with a longevity of up to several years. Upon receiving the same stimulation, memory cells could be activated rapidly and differentiate into memory T cells and effector T cells (Sprent and Surh, 2011; Mueller et al., 2013). Consistent with the previous finding that proliferating T cells experienced 3′UTR shortening (Gruber et al., 2014), we found that, during the conversion of naïve to memory cells (for both CD4^+^ and CD8^+^ T cells), several dynamic shortening APA events occurred, including those of the surface markers *CD5* and *CD28*; the immune functional genes *BCL2, TCF7, NFATC2IP, GOLT1B*, and *TMEM59*; and the protein ubiquitination-related genes *YPEL5, SUMO3*, and *UBE2G1. In vitro* validation in CD8^+^ T cells revealed a shortening trend, which was consistent with data analyses for most of these targets. However, in the CD8.CM cluster, only the shortening of *NFATC2IP* and *YPEL5* and the lengthening of *BCL2* were confirmed. Given that CD8.CM cells mainly reside in lymphoid organs (Sathaliyawala et al., 2013), this deviation could possibly originate from the biological difference between the major CD8.CM clusters in lymph nodes and residues in the blood.

Although a few novel insights were provided, several study limitations and future perspectives should be mentioned. First, because the selected computational method scAPA exhibited unsatisfying credibility and accuracy from intronic PAS, although we calculated both 3′UTR and intronic PASs, we mainly focused on 3′UTR APA in the subsequent analyses. Moreover, because of the lack of bed files of annotations in the scAPA pipeline, the PAS in upstream exonic and intergenic regions were not calculated. Furthermore, although the peripheral PAS switch was validated in CD8^+^ T cells from healthy humans, further experiments are needed to support the findings of the data analysis. Related mechanisms and the specific role of APA in lymphoid differentiation also require more in-depth studies. Third, although we used single-cell data to explore the landscape of APA in human lymphoid hematopoiesis, the APA dynamics were mainly analyzed at the cell-population level, rather than the single-cell level, because of the limitation of the scAPA analytical tool. Fourth, the position of the PAS given by scAPA was slightly rough. As stated by the authors of the scAPA pipeline (Shulman and Elkon, 2019), the resolution of PAS detection is an inherent limitation of the APA analysis using 3′ tag scRNA-seq data. The position difference between the detected PAS and the real PAS was mainly derived from the characteristics of the scRNA-seq method and the bioinformatics pipeline of scAPA. Nevertheless, to ensure the credibility of the detected PAS in this research, we assessed the quality via motif enrichment analysis and database benchmarking (Figure 2F; Supplementary Figure S1B–D). These results were comparable to the quality control reported by Shulman and Elkon (2019).

In summary, we provided a single-cell APA atlas of all lymphoid stages. This study also established a PAS resource and revealed the trend of 3′UTR shortening and enhanced intronic cleavage during the differentiation from very primitive HSPCs to the terminal clusters of B cells, T cells, NK cells, and pDCs. Specific dynamic-APA events were detected in each population. The APA patterns provide an improved definition of the differentiation stage and are possibly modulated by specific APA regulators. In the peripheral differentiation of CD4^+^ and CD8^+^ T cells, the proximal 3′UTR PASs of genes related to lymphoid function are preferred during the conversion of naïve to memory cells. Moreover, intronic APA possibly also played a role in the transition from HSPCs to terminally differentiated cells. Taken together, these findings have extended the knowledge of APA as an essential post-transcriptional mechanism and a unique molecular marker in hematopoiesis and the immune system.

## Materials and methods

### scRNA-seq data source and single-cell clustering

The single-cell transcriptome data were acquired from the Gene Expression Omnibus with the accession code GSE139369 (Granja et al., 2019). Six samples were collected from the bone marrow and peripheral blood of four healthy donors. Droplet-based cellular indexing of transcriptomes and epitopes by sequencing (CITE-seq) (Stoeckius et al., 2017) was used for sequencing. The authors simultaneously generated 10× Genomics 3′ scRNA-seq and antibody-derived tag sequencing data. scRNA-seq processing and quality control were performed by Granja and colleagues, as described in their article (Granja et al., 2019). The original BAM files were downloaded for APA analysis. The expression matrix of counts per million (CPM) was downloaded for single-cell reclustering and expression analyses.

The expression matrix of the lymphoid cells was extracted according to the initial cluster annotations in the data source, including HSCs; CMP.LMPP cells; common lymphoid progenitor 1 (CLP 1); common lymphoid progenitor 2 (CLP 2); Pre.B cells; B cells; plasma; CD4.N, CD4.M, CD8.N, CD8.CM, CD8.EM; NK cells; and pDCs. We reclustered these 22905 cells using the shared nearest neighbor (SNN) approach of Seurat (version 3.1.5) (Butler et al., 2018) based on the expression matrix. The clustering results were visualized using uniform manifold approximation and projection (UMAP) (Figure 1A; McInnes et al., 2020). In the clustering, we obtained 14 clusters and identified them using manually curated marker genes (Figure 1B), similar to the cluster annotation in the data-source article. To improve the accuracy of the cell identification, we only retained 20035 cells with a positive surface marker and an identity that was consistent with that reported in the data-source article (Supplementary Table S1). We further noticed that there were two CLP subpopulations, probably caused by the cell heterogeneity noted between the different donors (Supplementary Figure S1E). Therefore, we combined them into one CLP cluster. Moreover, because a small cell number may affect the credibility of PAS identification and differential analysis, we discarded the plasma cell cluster, which contained only 62 cells. Finally, 12 clusters of 19973 cells were subjected to the subsequent analysis (Figure 1C; Supplementary Table S2).

### Identification and quantification of the 3′UTR PAS

The scAPA (Shulman and Elkon, 2019) pipeline and R package (https://github.com/ElkonLab/scAPA) were used to identify and quantify 3′UTR PASs from the single-cell transcriptome data. To ensure the credibility of the data, we filtered out intronic peaks that met the following criteria: (i) a total of <10 CPMs over all 12 cell clusters; (ii) with a genomic sequence of eight consecutive adenines in the region from 10 nt to 140 nt downstream of the peak's 3′ edge.

### Quality control of the 3′UTR PAS

For motif enrichment analysis, position information of 3′UTR PASs was extracted from the scAPA object (the construction process is described below). The scAPA function *read_down.seq* was used to extract the 30-nt upstream and 150-nt downstream sequences of the peak 3′-end from the scAPA object. The MEME Suite tool DREME (Bailey, 2011) was used to enrich the consensus poly(A) signal motifs.

For database benchmarking, acknowledged human PASs of the 3′-most exon, 5′-most exon, internal exon, and single exon were downloaded from the PolyA_DB database (version 3.2) (exon.apps.wistar.org/PolyA_DB/v3, accessed on June 17, 2020) (Wang et al., 2018) and compared to our identified 3′UTR PASs.

### Identification, quantification, and quality control of the intronic PAS

Intronic PASs were identified in a manner similar to that used for 3′UTR identification, according to the methods reported by Shulman and Elkon (Shulman and Elkon, 2019). To ensure the credibility of the data, we filtered out intronic peaks that met the following criteria: (i) <5 CPMs in any cell cluster; (ii) a total of <10 CPMs over all 12 cell clusters; (iii) with a genomic sequence of seven consecutive adenines in the region from 1 nt to 200 nt downstream of the peak's 3′ edge.

### Differential APA analysis of 3′UTRs

We used the *calc_p_pui_mat* function of the scAPA package (Shulman and Elkon, 2019) to calculate pPUI, which represents the relative abundance of the proximal peak in each 3′UTR with multiple PASs. The calculation formula used for this was ${\mathrm{pPUI}} = {{\log }_2}{{( {{{C}_1} + 1} )}}/{{\langle {C + 1} \rangle }}$, the where ${{C}_1}$ is the read count of proximal peak and $\langle {C + 1} \rangle $ indicates the geometric mean of the read counts of all PASs associated with the given 3′UTR. The pseudo 1 was added to avoid zeros in the denominator and the log function.

To characterize the differential PAS usage between the different cell clusters, χ^2^ tests were performed on peak counts. Benjamini–Hochberg false discovery rate (FDR) correction was used to correct for multiple comparisons.

### Identification of cell type-specific and stage-specific APA events in 3′UTRs

To identify specifically shortened or lengthened 3′UTRs in a cell cluster of a specific differentiation stage, we used the *ROKU* function of the TCC package (Sun et al., 2013) to calculate the outliers of the pPUI matrix. In the output data frame, the value ‘−1’ indicated a low pPUI, and the corresponding 3′UTR was considered as having undergone lengthening; the value ‘1’ indicated a high pPUI, and the corresponding 3′UTR was considered as having undergone shortening; and the value ‘0’ indicated an intermediate pPUI, and the corresponding 3′UTR was considered as being nonspecific.

### Visualization of the read coverage

We first converted the BAM files of each cell cluster to the bigwig (bw) format using the deepTools (version 3.5.0) (Ramírez et al., 2014) function *bamCoverage* (-bs 10 –normalizeUsing CPM). The bw file was loaded onto the Integrative Genomics Viewer (Thorvaldsdóttir, Robinson and Mesirov, 2013) to browse the location and abundance of the peaks.

### GO enrichment analysis

GO enrichment analysis of human genes was performed using the online analysis tool Metascape (Zhou et al., 2019; http://metascape.org/gp/index.html#/main/step1).

### Single-cell clustering based on pPUI

The pPUI matrix was extracted from the scAPA results. We then clustered these single cells using the SNN approach of Seurat (version 3.1.5) (Butler et al., 2018), during which we kept the first 20 dimensions and used a resolution of 1.1. Finally, we visualized the results using UMAP (McInnes et al., 2020).

### Single-cell pseudotemporal ordering

Pseudotime analysis was performed using Monocle (version 2.0) (Trapnell et al., 2014) based on the single-cell RNA expression matrix. Cells exhibiting low gene expression levels were filtered out before calculation, and genes that were expressed in >10% of the cells were retained. We reduced the dimension using max_components = 2, method = ‘DDRTree’, and norm_method = ‘log’.

### Differential expression analysis of APA regulators

Differential expression analysis of scRNA-seq data was performed using the *FindMarkers* function of the R package Seurat (version 3.1.5) (Butler et al., 2018).

### Sample collection and human CD8^+^ T-cell isolation

Samples were collected from four healthy donors with informed consent in accordance with the Ethical Regulations of Peking Union Medical College. Individual information and health status are provided in Supplementary Table S4. Peripheral blood was obtained by venipuncture. Human CD8.N, CD8.CM, and CD8.EM cells were isolated using magnetic beads with the EasySep Human Naïve CD8^+^ T Cell and Memory CD8^+^ T-Cell Enrichment Kits, respectively (STEMCELL Technologies), according to the manufacturer's instructions.

### RNA extraction, reverse transcription, and qRT-PCR

Total RNA was extracted from the isolated human CD8^+^ T cells and converted to cDNA by reverse transcription. mRNA expression levels were measured using quantitative real-time polymerase chain reaction (qRT-PCR). The sequences of the primers and probes used are listed in Supplementary Table S5. The relative expression level of each transcript with different PAS was recorded as the ratio of the expression level of each transcript to that of the reference mRNA *ACTB* (encoding β-actin). Three replicates were performed for each transcript in each sample. The standard curves for the *ACTB* mRNA and other mRNAs showed good linearity between Cq values and the log of the samples.

### Analysis of the intronic PAS

In analogy to pPUI, iPUI was defined to characterize the relative usage of an intronic PAS versus the 3′UTR PAS of a given gene in a specific cell cluster. The calculation formula used here was ${\mathrm{iPUI}} = {{\log }_2}({{{{C}_{\mathrm{i}}} + 1}}/{{{{C}_{\mathrm{u}}} + 1}})$, where ${{C}_{\mathrm{i}}}$ is the read count mapped to the intronic peak and ${{C}_{\mathrm{u}}}$ indicates the sum of the read counts of all 3′UTR PASs. The pseudo 1 was added to avoid zeros in the denominator and the log function. A higher value of iPUI indicates a preference for intronic PASs. Differential intronic PAS cleavage between different cell clusters was analyzed based on peak counts using χ^2^ tests. Multiple comparisons were corrected with the FDR set to 5%. The enhancement or attenuation of the intronic cleavage of a given gene in a specific cell cluster or differentiation stage was analyzed. We used the *ROKU* function of the TCC package (Sun et al., 2013) to calculate the outliers of the iPUI matrix. In the output data frame, the value ‘1’ indicated a high iPUI, and the corresponding intronic cleavage was considered as enhanced; the value‘−1’ indicated a low iPUI and was considered as attenuated; and the value ‘0’ indicated an intermediate iPUI and was considered as nonspecific.

### Data availability

Data are available on request to the corresponding authors.

## Supplementary Material

mjae027_Supplemental_File

## Notes

**Author contributions:** J.Q., S.Y., and F.W. designed the study. J.Q. and S.Y. analyzed the bioinformatics data. J.L., X.W., Y.Z., and F.W. interpreted the results. J.Q. and Y.R. enrolled human donors and performed the validation experiments. J.Q. drafted the manuscript. F.W. and Y.Z. revised the manuscript. F.W. is the guarantor of the paper, taking responsibility for the integrity of the work as a whole, from inception to publication. All the authors read and approved the final manuscript.

## References

[bib1] Amon L., Lehmann C.H.K., Baranska A. et al. (2019). Transcriptional control of dendritic cell development and functions. Int. Rev. Cell Mol. Biol. 349, 55–151. 31759434 10.1016/bs.ircmb.2019.10.001

[bib2] Bailey T.L. (2011). DREME: motif discovery in transcription factor ChIP–seq data. Bioinformatics 27, 1653–1659. 21543442 10.1093/bioinformatics/btr261 PMC3106199

[bib3] Buenrostro J.D., Corces M.R., Lareau C.A. et al. (2018). Integrated single-cell analysis maps the continuous regulatory landscape of human hematopoietic differentiation. Cell 173, 1535–1548.e16. 29706549 10.1016/j.cell.2018.03.074 PMC5989727

[bib5] Butler A., Hoffman P., Smibert P. et al. (2018). Integrating single-cell transcriptomic data across different conditions, technologies, and species. Nat. Biotechnol. 36, 411–420. 29608179 10.1038/nbt.4096 PMC6700744

[bib6] Cheng L.C., Zheng D., Baljinnyam E. et al. (2020). Widespread transcript shortening through alternative polyadenylation in secretory cell differentiation. Nat. Commun. 11, 3182. 32576858 10.1038/s41467-020-16959-2 PMC7311474

[bib7] Chuvpilo S., Zimmer M., Kerstan A. et al. (1999). Alternative polyadenylation events contribute to the induction of NF-ATc in effector T cells. Immunity 10, 261–269. 10072078 10.1016/s1074-7613(00)80026-6

[bib8] Derti A., Garrett-Engele P., Macisaac K.D. et al. (2012). A quantitative atlas of polyadenylation in five mammals. Genome Res. 22, 1173–1183. 22454233 10.1101/gr.132563.111 PMC3371698

[bib9] Dunkle A., Dzhagalov I., Gordy C. et al. (2013). Transfer of CD8^+^ T cell memory using Bcl-2 as a marker. J. Immunol. 190, 940–947. 23269245 10.4049/jimmunol.1103481 PMC4366938

[bib10] Floro J., Dai A., Metzger A. et al. (2021). SDE2 is an essential gene required for ribosome biogenesis and the regulation of alternative splicing. Nucleic Acids Res. 49, 9424–9443. 34365507 10.1093/nar/gkab647 PMC8450105

[bib11] Granja J.M., Klemm S., McGinnis L.M. et al. (2019). Single-cell multiomic analysis identifies regulatory programs in mixed-phenotype acute leukemia. Nat. Biotechnol. 37, 1458–1465. 31792411 10.1038/s41587-019-0332-7 PMC7258684

[bib12] Gruber A.J., Zavolan M. (2019). Alternative cleavage and polyadenylation in health and disease. Nat. Rev. Genet. 20, 599–614. 31267064 10.1038/s41576-019-0145-z

[bib13] Gruber A.R., Martin G., Müller P. et al. (2014). Global 3′UTR shortening has a limited effect on protein abundance in proliferating T cells. Nat. Commun. 5, 5465. 25413384 10.1038/ncomms6465

[bib14] Hong Y., Xia Z., Sun Y. et al. (2023). A comprehensive pan-cancer analysis of the regulation and prognostic effect of coat complex subunit Zeta 1. Genes 14, 889. 37107648 10.3390/genes14040889 PMC10137353

[bib15] Hoque M., Ji Z., Zheng D. et al. (2013). Analysis of alternative cleavage and polyadenylation by 3′ region extraction and deep sequencing. Nat. Methods 10, 133–139. 23241633 10.1038/nmeth.2288 PMC3560312

[bib16] Hu H., Sun S.-C. (2016). Ubiquitin signaling in immune responses. Cell Res. 26, 457–483. 27012466 10.1038/cr.2016.40 PMC4822134

[bib17] Ji Z., Lee J.Y., Pan Z. et al. (2009). Progressive lengthening of 3′ untranslated regions of mRNAs by alternative polyadenylation during mouse embryonic development. Proc. Natl Acad. Sci. USA 106, 7028–7033. 19372383 10.1073/pnas.0900028106 PMC2669788

[bib18] Ji Z., Tian B. (2009). Reprogramming of 3′ untranslated regions of mRNAs by alternative polyadenylation in generation of pluripotent stem cells from different cell types. PLoS One 4 e8419. 20037631 10.1371/journal.pone.0008419 PMC2791866

[bib19] Karginov T.A., Ménoret A., Vella A.T. (2022). Optimal CD8^+^ T cell effector function requires costimulation-induced RNA-binding proteins that reprogram the transcript isoform landscape. Nat. Commun. 13, 3540. 35725727 10.1038/s41467-022-31228-0 PMC9209503

[bib20] Lee J., Robinson M.E., Ma N. et al. (2020). IFITM3 functions as a PIP3 scaffold to amplify PI3K signalling in B cells. Nature 588, 491–497. 33149299 10.1038/s41586-020-2884-6 PMC8087162

[bib21] Lianoglou S., Garg V., Yang J.L. et al. (2013). Ubiquitously transcribed genes use alternative polyadenylation to achieve tissue-specific expression. Genes Dev. 27, 2380–2396. 24145798 10.1101/gad.229328.113 PMC3828523

[bib22] Linterman M.A., Denton A.E., Divekar D.P. et al. (2014). CD28 expression is required after T cell priming for helper T cell responses and protective immunity to infection. eLife 3, e03180. 25347065 10.7554/eLife.03180 PMC4241536

[bib23] Liu T., Liu B., Liu Y. et al. (2021). Vesicle transporter GOLT1B mediates the cell membrane localization of DVL2 and PD-L2 and promotes colorectal cancer metastasis. Cancer Cell Int. 21, 287. 34059062 10.1186/s12935-021-01991-z PMC8166103

[bib24] McInnes L., Healy J., Melville J. (2020). UMAP: uniform manifold approximation and projection for dimension reduction. arXiv, 10.48550/arXiv.1802.03426

[bib25] Mitschka S., Mayr C. (2022). Context-specific regulation and function of mRNA alternative polyadenylation. Nat. Rev. Mol. Cell Biol. 23, 779–796. 35798852 10.1038/s41580-022-00507-5 PMC9261900

[bib26] Mueller S.N., Gebhardt T., Carbone F.R. et al. (2013). Memory T cell subsets, migration patterns, and tissue residence. Annu. Rev. Immunol. 31, 137–161. 23215646 10.1146/annurev-immunol-032712-095954

[bib27] Murphy K., Weaver C. (2016). Janeway's Immunobiology (9th edn). New York, USA: Garland Science, Taylor & Francis Group.

[bib28] Ramírez F., Dündar F., Diehl S. et al. (2014). deepTools: a flexible platform for exploring deep-sequencing data. Nucleic Acids Res. 42, W187–W191. 24799436 10.1093/nar/gku365 PMC4086134

[bib29] Sandberg R., Neilson J.R., Sarma A. et al. (2008). Proliferating cells express mRNAs with shortened 3′ untranslated regions and fewer microRNA target sites. Science 320, 1643–1647. 18566288 10.1126/science.1155390 PMC2587246

[bib30] Sathaliyawala T., Kubota M., Yudanin N. et al. (2013). Distribution and compartmentalization of human circulating and tissue-resident memory T cell subsets. Immunity 38, 187–197. 23260195 10.1016/j.immuni.2012.09.020 PMC3557604

[bib31] Shulman E.D., Elkon R. (2019). Cell type-specific analysis of alternative polyadenylation using single-cell transcriptomics data. Nucleic Acids Res. 47, 10027–10039. 31501864 10.1093/nar/gkz781 PMC6821429

[bib32] Singh I., Lee S.-H., Sperling A.S. et al. (2018). Widespread intronic polyadenylation diversifies immune cell transcriptomes. Nat. Commun. 9, 1716. 29712909 10.1038/s41467-018-04112-z PMC5928244

[bib33] Sommerkamp P., Altamura S., Renders S. et al. (2020). Differential alternative polyadenylation landscapes mediate hematopoietic stem cell activation and regulate glutamine metabolism. Cell Stem Cell 26, 722–738.e7. 32229311 10.1016/j.stem.2020.03.003

[bib34] Sommerkamp P., Cabezas-Wallscheid N., Trumpp A. (2021). Alternative polyadenylation in stem cell self-renewal and differentiation. Trends Mol. Med. 27, 660–672. 33985920 10.1016/j.molmed.2021.04.006

[bib35] Sprent J., Surh C.D. (2011). Normal T cell homeostasis: the conversion of naïve cells into memory-phenotype cells. Nat. Immunol. 12, 478–484. 21739670 10.1038/ni.2018 PMC3434123

[bib36] Stoeckius M., Hafemeister C., Stephenson W. et al. (2017). Simultaneous epitope and transcriptome measurement in single cells. Nat. Methods 14, 865–868. 28759029 10.1038/nmeth.4380 PMC5669064

[bib37] Sun J., Nishiyama T., Shimizu K. et al. (2013). TCC: an R package for comparing tag count data with robust normalization strategies. BMC Bioinformatics 14, 219. 23837715 10.1186/1471-2105-14-219 PMC3716788

[bib38] Thorvaldsdóttir H., Robinson J.T., Mesirov J.P. (2013). Integrative Genomics Viewer (IGV): high-performance genomics data visualization and exploration. Brief. Bioinform. 14, 178–192. 22517427 10.1093/bib/bbs017 PMC3603213

[bib39] Tian B., Graber J.H. (2012). Signals for pre-mRNA cleavage and polyadenylation. Wiley Interdiscip. Rev. RNA 3, 385–396. 22012871 10.1002/wrna.116 PMC4451228

[bib40] Tian B., Manley J.L. (2017). Alternative polyadenylation of mRNA precursors. Nat. Rev. Mol. Cell Biol. 18, 18–30. 27677860 10.1038/nrm.2016.116 PMC5483950

[bib41] Tian B., Pan Z., Lee J.Y. (2007). Widespread mRNA polyadenylation events in introns indicate dynamic interplay between polyadenylation and splicing. Genome Res. 17, 156–165. 17210931 10.1101/gr.5532707 PMC1781347

[bib42] Trapnell C., Cacchiarelli D., Grimsby J. et al. (2014). The dynamics and regulators of cell fate decisions are revealed by pseudotemporal ordering of single cells. Nat. Biotechnol. 32, 381–386. 24658644 10.1038/nbt.2859 PMC4122333

[bib43] Wang R., Nambiar R., Zheng D. et al. (2018). PolyA_DB 3 catalogs cleavage and polyadenylation sites identified by deep sequencing in multiple genomes. Nucleic Acids Res. 46, D315–D319. 29069441 10.1093/nar/gkx1000 PMC5753232

[bib44] Xia Y., Midoun S.Z., Xu Z. et al. (2015). Heixuedian (heix), a potential melanotic tumor suppressor gene, exhibits specific spatial and temporal expression pattern during Drosophila hematopoiesis. Dev. Biol. 398, 218–230. 25530181 10.1016/j.ydbio.2014.12.001

[bib45] Yang X., Tong Y., Liu G. et al. (2022). scAPAatlas: an atlas of alternative polyadenylation across cell types in human and mouse. Nucleic Acids Res. 50, D356–D364. 34643729 10.1093/nar/gkab917 PMC8728290

[bib46] Ye C., Lin J., Li Q.Q. (2020). Discovery of alternative polyadenylation dynamics from single cell types. Comput. Struct. Biotechnol. J. 18, 1012–1019. 32382395 10.1016/j.csbj.2020.04.009 PMC7200215

[bib47] Zhang C., Cheng M., Dong N. et al. (2022). General transcription factor IIF polypeptide 2: a novel therapeutic target for depression identified using an integrated bioinformatic analysis. Front. Aging Neurosci. 14, 918217. 35711908 10.3389/fnagi.2022.918217 PMC9197343

[bib48] Zhang J., Lyu T., Cao Y. et al. (2021). Role of TCF-1 in differentiation, exhaustion, and memory of CD8^+^ T cells: a review. FASEB J. 35, e21549. 33913198 10.1096/fj.202002566R

[bib49] Zhou Y., Zhou B., Pache L. et al. (2019). Metascape provides a biologist-oriented resource for the analysis of systems-level datasets. Nat. Commun. 10, 1523. 30944313 10.1038/s41467-019-09234-6 PMC6447622

