# Supplementary material for: Single-cell landscape of alternative polyadenylation in human lymphoid hematopoiesis
Source: J Mol Cell Biol. 2024 Jul 9;16(7):mjae027. doi: 10.1093/jmcb/mjae027 (PMC11736434; doi:10.1093/jmcb/mjae027)
Supplement: mjae027_Supplemental_File [file mjae027_supplemental_file.pdf]

Supplementary File for

Single-Cell Landscape of Alternative Polyadenylation in Human Lymphoid Hematopoiesis

Short title: Single-cell lymphoid APA atlas

Author: Jiaqi Qiang[1,2,3#], Shan Yu[1,2,4#], Jun Li[5#], Yu Rong[1,2], Xiaoshuang Wang[1,2*], Yong Zhu[6*], Fang Wang[1,2*]

# Co-first authorship: Jiaqi Qiang, Shan Yu, Jun Li

Author Affiliation:

[1] State Key Laboratory of Medical Molecular Biology, Department of Biochemistry and Molecular Biology, Institute of Basic Medical Sciences, Chinese Academy of Medical Sciences, School of Basic Medicine, Peking Union Medical College, Beijing 100005, China

[2] The Key Laboratory of RNA and Hematopoietic Regulation, Chinese Academy of Medical Sciences, Beijing 100005, China

[3] Eight-Year Program of Clinical Medicine, Chinese Academy of Medical Sciences and Peking Union Medical College, Beijing 100730, China

[4] Key Laboratory of Digital Technology in Medical Diagnostics of Zhejiang Province, Hangzhou 310030, China

[5] Department of Cardiovascular Medicine, Chongqing Emergency Medical Center, Chongqing University Central Hospital, Chongqing 400014, China

[6] Institute of Life Sciences, Chongqing Medical University, Chongqing 400016, China

Correspondence to: Fang Wang, Institute of Basic Medical Sciences, Peking Union Medical College, No. 5 San Tiao Dongdan, Beijing 100005, China; Phone: 8610-6915-6423; Fax: 8610-6915-6423; Email: wo_wfang@hotmail.com. Yong Zhu, Institute of Life Sciences, Chong Qing Medical University, #1 Medical College Road, Chongqing 400016, China; Email: yongz59@cqmu.edu.cn. Xiaoshuang Wang, Institute of Basic Medical Sciences, Peking Union Medical College, No. 5 San Tiao Dongdan, Beijing 100005, China; Email: cattle1131@163.com.

Supplementary Table S1. Labels and filtering of single cells in the re-clustering.

Cluster	Labels in the source article	Labels in the re-clustering [a]	Overlap [b]	Positive surface marker
HSC	1425	1616	1340	1111
CMP.LMPP	2260	2173	1971	1850
CLP.1	903	779	714	655
CLP.2	377	401	365	320
Pre.B	710	713	700	700
B	1711	1680	1676	1676
Plasma	62	102	62	62
CD8.N	1521	1510	1357	1357
CD4.N1	2470	4802	4426	4426
CD4.N2	2364			
CD4.M	3539	3781	3182	3182
CD8.EM	796	788	712	712
CD8.CM	2080	2157	1665	1665
NK	2143	1859	1810	1810
pDC	544	544	541	509
Total	22905	22905	20521	20035

[a] Represents the number of cells annotated in the re-clustering.

[b] Represents the number of cells with identical annotations in the source article and re-clustering.

Abbreviations: HSC, hematopoietic stem cell; CMP.LMPP, common myeloid progenitor/lymphoid-primed multipotent progenitor; CLP, common lymphoid progenitor; CD4.N, $CD4^+$ naïve cell; CD4.M, $CD4^+$ memory cell; CD8.N, $CD8^+$ naïve cell; CD8.CM, $CD8^+$ central memory cell; CD8.EM, $CD8^+$ effector memory cell; NK, natural killer cell; pDC, plasmacytoid dendritic cell.

Supplementary Table S2. Cluster and number of cells applied in the final analysis.

Cluster	Number
HSC	1111
CMP.LMPP	1850
CLP	975
Pre.B	700
B	1676
CD4.N	4426
CD4.M	3182
CD8.N	1357
CD8.CM	1665
CD8.EM	712
NK	1810
pDC	509
Total	19973

Abbreviations: HSC, hematopoietic stem cell; CMP.LMPP, common myeloid progenitor/lymphoid-primed multipotent progenitor; CLP, common lymphoid progenitor; CD4.N, $CD4^+$ naïve cell; CD4.M, $CD4^+$ memory cell; CD8.N, $CD8^+$ naïve cell; CD8.CM, $CD8^+$ central memory cell; CD8.EM, $CD8^+$ effector memory cell; NK, -natural killer cell; pDC, plasmacytoid dendritic cell.

Supplementary Table S3. Functional annotations for genes associated with lymphoid hematopoiesis and immune function in health and disease in populations of which the number of cell-type-specific 3'UTR APA events was insufficient for the GO enrichment.

Cluster	APA event	Gene	Functional annotations
HSC	shortening	*PGM3*	Immunodeficiency.
CMP.LMPP	shortening	*CD164*	Encodes a transmembrane salivary acid mucin and cell adhesion molecule that regulates proliferation, adhesion and migration of hematopoietic progenitor cells. High expression of observed in Sezary syndrome (blood cancer).
CLP	shortening	*VPS36*	Associated infectious diseases and vesicle-mediated transport pathways.
Pre.B	shortening	*CASP10*	Associated Type IIA autoimmune lymphoproliferative syndrome, non-Hodgkin's lymphoma and cancer.
Pre.B	shortening	*BCL2*	Related to Lymphocytes.
Pre.B	shortening	*RAB7A*	Involved in cellular vacuolization of the VacA cytotoxin of Helicobacter pylori
Pre.B	shortening	*MALT1*	This protein is a component of the CARMA1-BCL10-MALT1 (CBM) signalosome that triggers NF-κB signaling and lymphocyte activation upon antigen receptor stimulation. Mutations in this gene result in immunodeficiency 12 (IMD12). This gene has been found to be repeatedly rearranged in chromosomal translocations with other genes in mucosa-associated lymphoid tissue lymphomas, including a t(11; 18)(q21; q21) translocation to the baculovirus IAP repeat-sequence-containing protein 3 (also known as inhibitor of apoptosis 2) locus [BIC3(API2)-MALT1], as well as a t(11; 18)(q21; q21) translocation to the immunoglobulin heavy chain locus (IGH- MALT1) with the t(14; 18) (q32; q21) translocation.
Pre.B	shortening	*STAT5B*	TCR signaling; associated with APL
B	shortening	*KPNA1*	Related diseases include Venezuelan equine encephalitis and encephalitis
CD4.N	shortening	*ICOSLG*	Expected to be involved in the positive regulation of the T cell receptor signaling pathway and interleukin-4 production. ICOSLG (inducible T cell costimulator ligand) is a protein coding gene. Diseases associated with ICOSLG include combined immunodeficiency and B-cell deficiency.

			Affiliated pathways include CD28 signaling in helper t cells and NFAT in the immune response.
CD4.M	shortening	*GSK3B*	Involved in energy metabolism, inflammation, endoplasmic reticulum stress, mitochondrial dysfunction and apoptotic pathways.
CD8.N	shortening	*BTN2A2*	This gene is located in a cluster of seven genes on chromosome 6 and belongs to the expanding B7/eosinophilic butyrophilic acid protein-like gene cluster, a subset of the immunoglobulin gene superfamily. Its associated pathways include the innate immune system and class I MHC-mediated antigen processing and presentation.
CD8.N	shortening	*TAB3*	Function in the NF-κB signaling pathway. The encoded protein and the similar and functionally redundant protein MAP3K7IP2/TAB2 form a ternary complex with the protein kinases MAP3K7/TAK1 and TRAF2 or TRAF6 in response to stimulation by the pro-inflammatory cytokines TNF or IL-1. Subsequent MAP3K7/TAK1 kinase activity triggers a signaling cascade leading to activation of the NF-κB transcription factor.
CD8.EM	shortening	*MAVS*	Encodes an intermediate protein necessary in the virus-triggered interferon signaling pathway. It is required for the activation of transcription factors that regulate interferon expression and contributes to antiviral innate immunity. Related disease: oral diseases and hepatitis. Related pathways: DDX58/ ifih1-mediated interferon- α/β induction and SARS-CoV-2 infection.
CD8.EM	shortening	*CYLD*	Related pathway: TNF signaling and DDX58/IFIH1-mediated induction of interferon-α/β.
CD8.EM	shortening	*PLSCR1*	The encoded protein is implicated in gene regulation and interferon-induced antiviral responses.
CD8.EM	shortening	*TRIM5*	May play a role in restricting retroviruses. Related disease: immunodeficiency diseases and human immunodeficiency virus type 1. Related pathway: interferon signaling and cytokine signaling in the immune system.
NK	shortening	*SPPL3*	Involved in several processes including the T cell receptor signaling pathway. Related disease: plasmodium ovale malaria and sulfa allergy.
NK	shortening	*TNFSF13B*	This cytokine is expressed in B-cell lineage cells and acts as a potent B-cell activator. It has also been shown to play an important role in B cell

			proliferation and differentiation. Related disease: Sjogren syndrome and autoimmune diseases. Related pathway: MIF-mediated glucocorticoid regulation pathway and TGF-β pathway.
NK	shortening	*NR3C1*	Involved in inflammatory response, cell proliferation and target tissue differentiation.
pDC	shortening	*UBA5*	Related pathways: class I MHC-mediated antigen processing and presentation and the innate immune system.
pDC	shortening	*TUSC2*	Predicted to be involved in the inflammatory response and regulation of mitochondrial membrane potential. Prediction acts upstream of or within several processes, including natural killer cell differentiation; neutrophil-mediated killing of gram-negative bacteria; and regulation of cytokine production.
pDC	shortening	*ZBTB7B*	Encodes a zinc finger-containing transcription factor that is a key regulator of immature T cell precursor lineage commitment. It is necessary and sufficient for CD4 lineage commitment, while its absence results in CD8 commitment.
pDC	shortening	*PBXIP1*	Related diseases: leukemia.
pDC	lengthening	*TOX*	Contains an HMG-box DNA-binding structural domain that may function to regulate T cell development.
pDC	lengthening	*FUT10*	Related pathways: blood type system biosynthesis and glycosaminoglycan metabolism.
CD8.EM	lengthening	*ARID4B*	Plays a role in a variety of cellular processes including proliferation, differentiation, apoptosis, tumorigenesis, and cell fate determination. The gene product is recognized by IgG antibodies isolated from breast cancer patients and appears to be a molecular marker associated with a wide range of human malignancies. Its associated pathways include RNA polymerase I promoter opening and infectious diseases.
CD8.EM	lengthening	*PUM2*	Cell differentiation related. Related pathways: protein metabolism and class I MHC-mediated antigen processing and presentation.
CD8.CM	lengthening	*SYK*	Widely expressed in hematopoietic cells and involved in coupling activated immune receptors to downstream signaling events that mediate a variety of cellular responses, including proliferation, differentiation, and phagocytosis. Related disease: immunodeficiency with systemic inflammation and

			arthritis. Related pathways: actin dynamics that regulate phagocytic cup formation and ADORA2B-mediated production of anti-inflammatory cytokines.
CD8.N	lengthening	*PSMC2*	This subunit may compete with PSMC3 for binding to HIV proteins to regulate interactions between viral proteins and transcription complexes.
CD8.N	lengthening	*PTPRC*	An important regulator of antigen receptor signaling in T cells and B cells. It acts through direct interaction with components of the antigen receptor complex or through activation of various Src family kinases required for antigen receptor signaling. Related disease: immunodeficiency. Related pathways: camp-dependent PKA and activation of the innate immune system.
CD8.N	lengthening	*HSPA9*	Related disease: Even-Plus syndrome, anemia, and sideroblastic anemia. Related pathways: interleukin-12 family signaling and cellular response to stimuli.
CD8.N	lengthening	*NCBP2*	Related pathways: formation of the HIV extension complex in the absence of HIV Tat and translocation of SLBP-independent mature mRNA.
CD8.N	lengthening	*IFIT2*	Involved in the negative regulation of protein binding activity; positive regulation of apoptotic processes; and response to viruses. Relevant pathways: interferon-mediated signaling pathways overview and immune system cytokine signaling pathways.
CD4.M	lengthening	*IGHG3*	Enables antigen-binding activity and immunoglobulin receptor-binding activity. Located in blood particles and cellular exosomes. Related diseases: heavy chain disease. Related pathways: immune response, NK cell CD16 signaling pathway, heparin-induced thrombocytopenia pathway, and adverse drug reactions.
CD4.N	lengthening	*PLCG2*	Related disease: autoinflammatory diseases, antibody deficiency, immune dysregulation, and familial cold autoinflammatory syndrome. Related pathways: prolactin signaling and ADORA2B-mediated production of anti-inflammatory cytokines.
B	lengthening	*OTULIN*	Encodes an enzyme specifically recognizes and removes M1 (Met1) linkages or linear ubiquitin chains from protein substrates. The linear ubiquitin chain is known to regulate the NF-κB signaling pathway in the context of immunity and inflammation. Related disease: autoinflammatory diseases, panniculitis, dermatologic syndromes,

			immunodeficiency, invasive Staphylococcus aureus infections. Related pathways: TNF signaling and protein metabolism.
B	lengthening	*FYB1*	Encodes an adapter for the FYN protein and LCP2 signaling cascade in T cells. The encoded protein is involved in platelet activation and controls interleukin-2 expression. Related disease: thrombocytopenia and congenital autosomal recessive microthrombocytopenia. Related pathways: TCR signaling and cell junction organization.
Pre.B	lengthening	*CTSC*	Appears to be a central coordinator of the activation of many serine proteases in the immune system. Related pathways: translocation to the Golgi and its subsequent modification and the innate immune system.
Pre.B	lengthening	*SELL*	The gene product is required for the binding and subsequent rolling of leukocytes on endothelial cells, promoting their migration to secondary lymphoid organs and sites of inflammation. Related diseases: immunoglobulin A nephropathy, cryptococcosis. Related pathways: the innate immune system and class I MHC-mediated antigen processing and presentation.
CLP	lengthening	*IL4R*	The alpha chain of the interleukin 4 receptor.
CMP.LMPP	lengthening	*RUNX1*	Associated with the development of normal hematopoiesis; leukemia.
CMP.LMPP	lengthening	*ZBTB24*	Predicted to act upstream or within hematopoietic progenitor cell differentiation.
HSC	lengthening	*TCF3*	Plays a key role in lymphangiogenesis and encodes a protein that is essential for the development of B and T lymphocytes. Deletion of this gene or reduced activity of the encoded protein may play a role in lymphoid malignancies.
HSC	lengthening	*ASXL1*	Mutations in this gene linked to myelodysplastic syndromes and chronic granulomonocytic leukemia.
HSC	lengthening	*WDR1*	Related diseases: immunodeficiency, Thrombocytopenia Syndrome. Related pathways: response to elevated platelet cytoplasmic Ca^{2+}.

Supplementary Table S4. Health information of donors.

Sample	Sex	Age	Past medical history
0	Female	25	None
1	Male	27	None
2	Male	27	None
3	Male	27	None

Supplementary Table S5. Sequences of primers used in RT-qPCR.

Gene	Primer	Sequence
BCL2	F1	GCCTCTGTTTGATTTCTCCTGG
BCL2	R1	CCTGCAGCTTTGTTTCATGG
BCL2	F3	TCCGACCACTAATTGCCAAG
BCL2	R3	GAATGAACACCTTCTCCCCAG
BCL2	F4	GCTTATGAAGGTTCCAATCAGATC
BCL2	R4	AAACTTCCAACTCCCTGATCC
CD28	F1	CTCCTGCACAGTGACTACATG
CD28	R1	GGTCATTTCCTATCCAGAGCAG
CD28	F5	ACAGTGGTAGGAGCAATGC
CD28	R5	ATGCTTAACCCAAATAGGTGAGC
CD5	F1	GAATACAGCCAACCTCCCAG
CD5	R1	GATCCCAGTTCTTTACAGCCTC
CD5	F2	TCCTTTCTCCACGCCATTTG
CD5	R2	ACTCCCTTTTGCTCTTCCAG
GOLT1B	F1	CCTTTGATAGGCATGATCTTCG
GOLT1B	R1	ACTTTATCTACAAATGATCTAATTCCAGG
GOLT1B	F2	GGGTTTGATAATCTCCCAGTCC
GOLT1B	R2	GCAGTCCCAAACTCACATTTG
NFATC2IP	F1	CTAAAGACCCTCATGTCCCAC
NFATC2IP	R1	GTCATTCTCCCCAAGTCCAG
NFATC2IP	F4	ATGAGCCAGTTTTCTCAGTAATGTC
NFATC2IP	R4	AGCTGTACCATGTCGTGGAT
SUMO3	F1	GATCAGATTCAGGTTCGACGG
SUMO3	R1	CCTCTAGAAACTGTGCCCTG
SUMO3	F3	GCTTGAAATCTTGCTGAAATGTTC
SUMO3	R3	AACACAACAGAGCCAACAAAC
TCF7	F1	ACACAGGTACAGCAACAGG
TCF7	R1	GAGATGACGTTAACAGGACCC
TCF7	F2	ACCTTCTCTACCCATCTCCC
TCF7	R2	GCTTTGTGCTTGTGACCATG
TMEM59	F1	AGGAGTTGTACGCATGTCAG
TMEM59	R1	TGTCTCAGTTCAGCGAATGG
TMEM59	F2	AGAGGTGGATGTTTGGGATTG
TMEM59	R2	CCATTCCTGAGCAGAAGCATC
UBE2G1	F1	AGTTGCCCGCTGTGTAAG
UBE2G1	R1	ACAGAAGTCCAGCAATAGGTG
UBE2G1	F3	CCTAAACTTGTGCCAACCATC
UBE2G1	R3	TCATCTATCCCATACAAGCACAG
YPEL5	F1	GCAATAGCAAACTGGGATGG
YPEL5	R1	GGAGACCTGGGAAAAGATGG

YPEL5	F2	AATCTGCATCTGTTTTAGAAAAGGG
YPEL5	R2	AGATCTAGGCCTTTGATGGAAAC

Abbreviations: F, forward; R, reverse.

Supplementary Figures

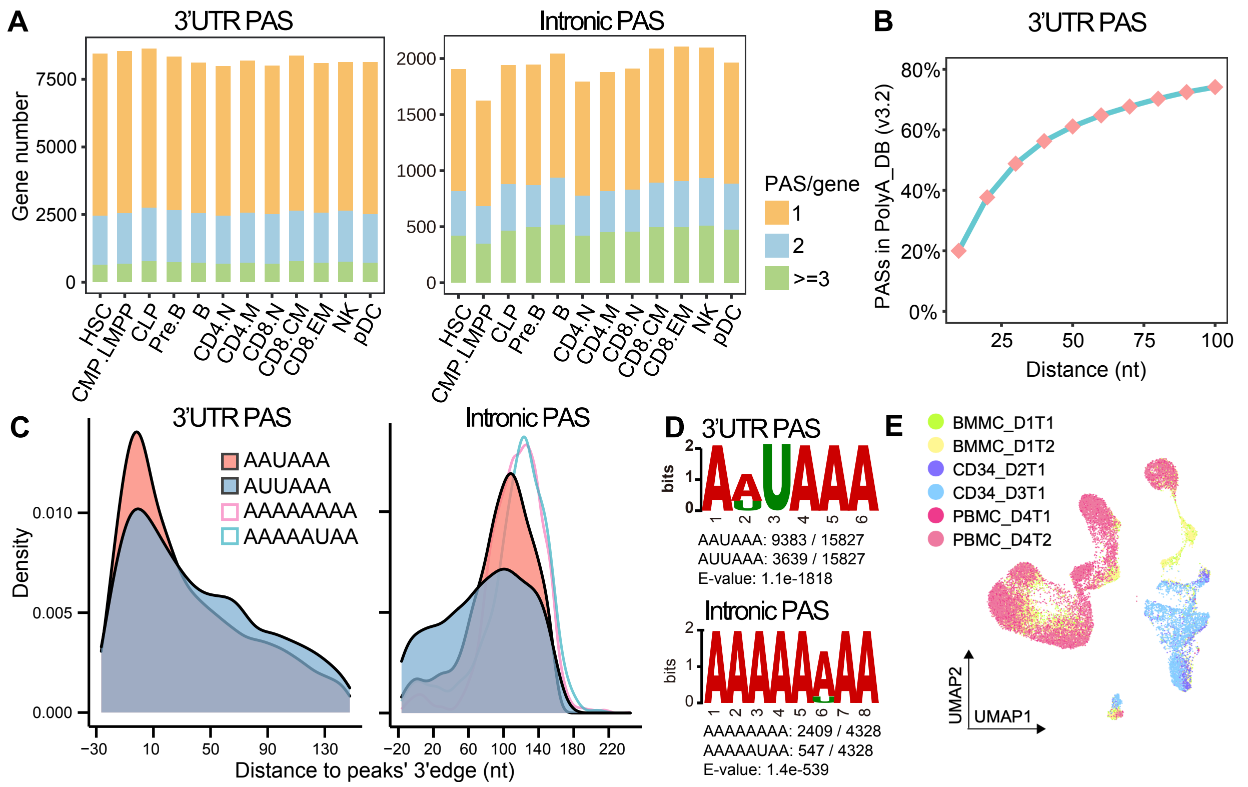

Supplementary Figure S1. Single-cell clustering and PAS identification during lymphocyte differentiation. (**A**) Quantitative distribution of PAS per gene in each population during lymphoid differentiation. (**B**) Benchmarking of the identified PASs. The cumulative curve showed the percentage of identified PASs within 100 nt from the 3′UTR PAS in the PolyA_DB database (version 3.2). (**C**) Distribution density of the canonical poly(A) signals AAUAAA and AUUAAA near PASs. (**D**) Motif enrichment of the sequence surrounding the identified PASs. (**E**) Sample source of the lymphocytes used in the single-cell clustering analysis. Abbreviations: BMMC_D1T1 & BMMC_D1T2, replicates 1 & 2 of bone-marrow mononuclear cells from donor 1; CD34_D2T1 & CD34_D3T1, replicates of CD34^{+}-enriched bone-marrow cells from donors 2 & 3; PBMC_D4T1 & PBMC_D4T2, replicates 1 & 2 of peripheral blood mononuclear cells from donor 4.

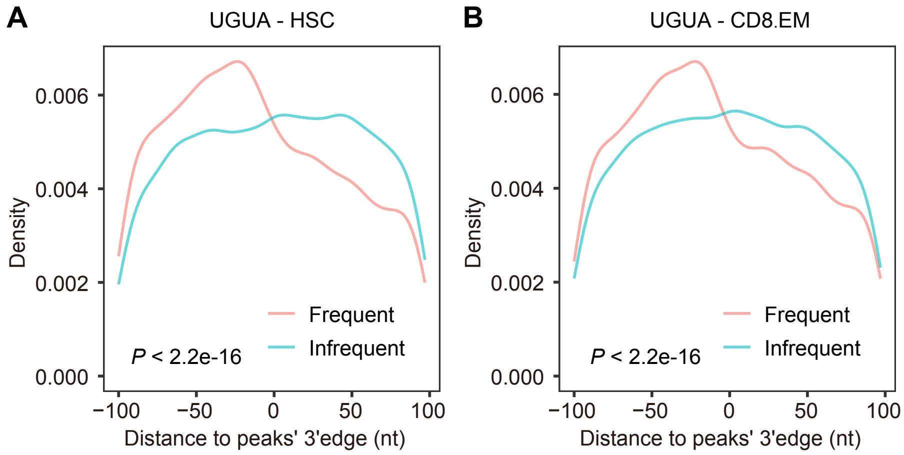

Supplementary Figure S2. The global APA analysis showed a trend of 3′UTR shortening during the development of lymphocytes. (**A**) Density distribution of the PAS enhancer UGUA near frequent and infrequent PASs in each 3′UTR with dynamic-APA events in HSCs. The Kolmogorov–Smirnov test was used to demonstrate the distribution difference between frequent PASs and infrequent PASs in the region located 100 nt upstream and 100 nt downstream of the 3′-end of the identified PASs. Frequent, PAS with the highest expression; infrequent, PAS with the lowest expression. (**B**) Density distribution of the PAS enhancer UGUA near frequent and infrequent PASs in each 3′UTR with dynamic-APA events in CD8.EM.

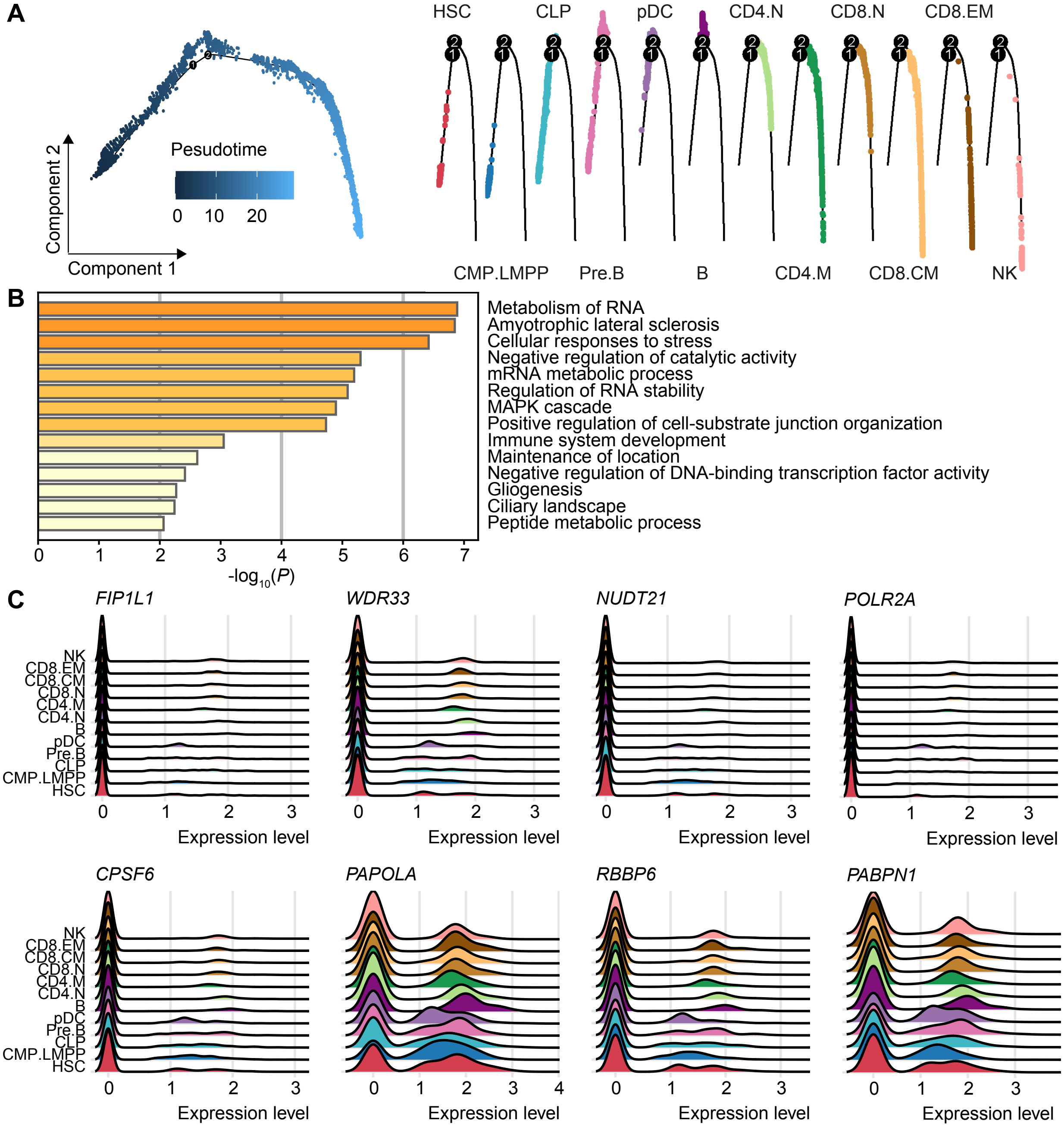

Supplementary Figure S3. APA reveals the differentiation stage in lymphocyte development. (**A**) Pseudotime analysis showing the differentiation trajectory based on RNA expression. Left, the pseudotime value of each cell (larger values indicated a higher degree of differentiation); right, display cell distribution on the trajectory map in each population, respectively. (**B**) GO enrichment analysis of specific 3′UTR-lengthening genes in HSPCs; (**C**) Ridge plots showing the mRNA expression of eight highly expressed core APA regulators in lymphoid populations.

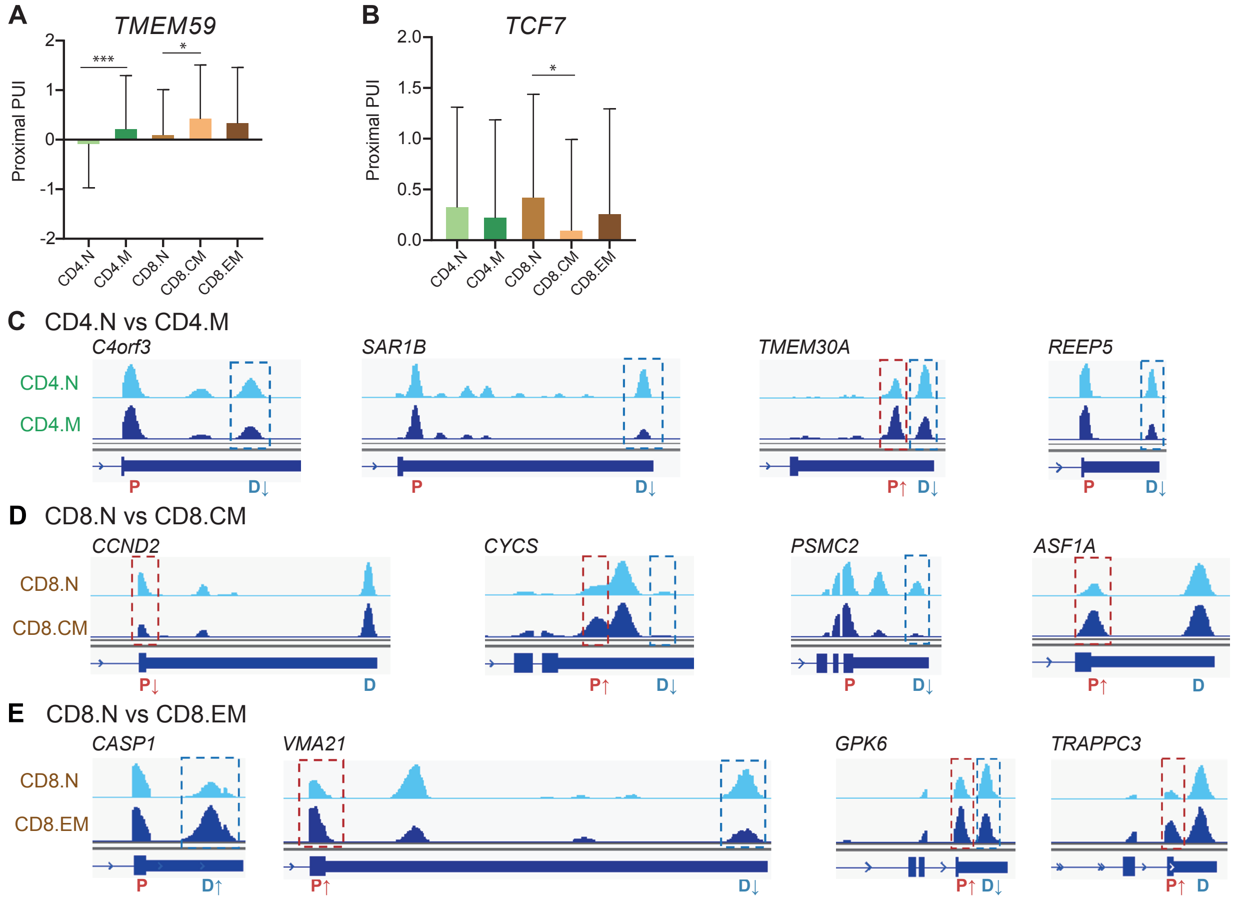

Supplementary Figure S4. Supplementary case visualization of the differential APA analysis during the peripheral differentiation of T cells. (A) Boxplot of the *t*-test for pPUI at the single-cell level of *TMEM59* (**P* < 0.05, ***P* < 0.01, ****P* < 0.001). **(B)** Boxplot of the *t*-test for pPUI at the single-cell level of *TCF7* (**P* < 0.05, ***P* < 0.01, ****P* < 0.001). **(C)** Cases with significant peak changes in CD4.N vs CD4.M cells. **(D)** Cases with significant peak changes in CD8.N vs. CD8.CM cells. **(E)** Cases with significant peak changes in CD8.N vs. CD8.EM cells.

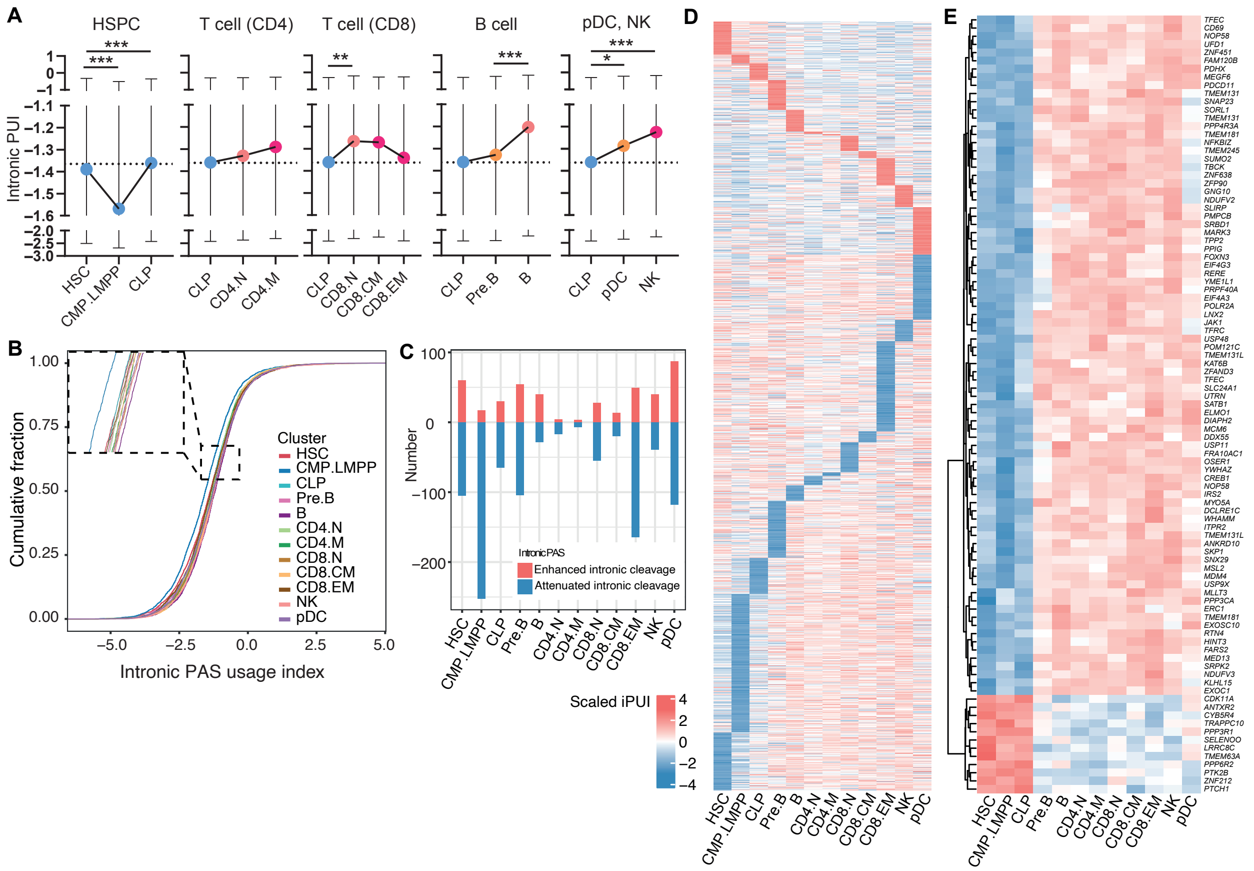

Supplementary Figure S5. Intronic APA trend during lymphoid differentiation. (**A**) The average iPUI of the 3,021 genes exhibiting significant intronic APA in each lymphoid cell population. A *t*-test was performed to determine the iPUI difference between adjacent populations in the differentiation trajectory (*P < 0.05, **P < 0.01, ***P < 0.001). (**B**) Cumulative distribution curve of the iPUI value of 3,021 genes with significant intronic APA in 12 lymphoid cell populations. The square on the upper left indicates partial magnification. The statistical significance of the differences between adjacent clusters during differentiation: HSCs vs. CMP.LMPP, P < 0.001; CMP.LMPP vs. CLP, P < 0.001; CLP vs. Pre.B, P = 0.227; CLP vs. CD4.N, P = 0.254; CLP vs. CD8.N, P < 0.001; CLP vs. NK, P < 0.001; CLP vs. pDC, P = 0.006; Pre.B vs. B, P < 0.001; CD4.N vs. CD4.M, P = 0.128; CD8.N vs. CD8.CM, P = 0.840; CD8.N vs. CD8.EM, P = 0.466. (**C**) Number of enhanced and attenuated intronic cleavage events in each lymphoid cell cluster. (**D**) iPUI heatmap of specific enhanced/attenuated intronic cleavage PASs in the 12 lymphoid cell populations. Red, enhanced intronic cleavage; blue, attenuated intronic cleavage. (**E**)

iPUI heatmap of stage-specific APA events. Red, specifically enhanced intronic cleavage; blue, specifically attenuated intronic cleavage.
